# Translational molecular imaging and drug development in multiple sclerosis

**DOI:** 10.7150/thno.119559

**Published:** 2026-01-01

**Authors:** Daniel Tay, Hazem Ahmed, Alyaa Dawoud, Mohamed Salam, Luca Gobbi, Uwe Grether, Martin R. Edelmann, Matthias B. Wittwer, Ludovic Collin, Kenneth Atz, James Keaney, Maude Giroud, Alexia Rossi, Antonio Giulio Gennari, Gennaro Pagano, Neil John Parrot, Muhamed Barakovic, Axel Rominger, Catherine Gebhard, Simon M. Ametamey, Amit M. Saindane, Steven H. Liang, Achi Haider

**Affiliations:** 1 Department of Biology and Applied Sciences, ETH Zurich, Otto-​Stern-Weg 1, 8093 Zurich, Switzerland.; 2 Center for Radiopharmaceutical Sciences ETH-PSI-USZ, Institute of Pharmaceutical Sciences ETH, Vladimir-Prelog-Weg 4, 8093 Zurich, Switzerland; 3 Biochemistry Department, Faculty of Pharmacy and Biotechnology, German University in Cairo, 11835 Cairo, Egypt.; 4 Department of Nuclear Medicine, University Hospital Zurich, University of Zurich, Zurich, Switzerland.; 5 Pharma Research and Early Development, Roche Innovation Center Basel, F. Hoffmann-La Roche Ltd., Basel, Switzerland.; 6 Department of Nuclear Medicine, Inselspital, Bern University Hospital, University of Bern, Freiburgstr. 18, 3010 Bern, Switzerland.; 7 Department of Cardiology, University Hospital Bern, Bern, Switzerland.; 8 Department of Radiology and Imaging Sciences, Emory University, 1364 Clifton Road, Atlanta, GA 30322, USA.; 9 Department of Radiology, Division of Nuclear Medicine and Molecular Imaging Massachusetts General Hospital and Harvard Medical School, 55 Fruit Street, Boston, MA 02114, USA.

**Keywords:** translational molecular imaging, multiple sclerosis, demyelination, positron emission tomography (PET), neuroinflammation, tracer development, drug development

## Abstract

Multiple sclerosis (MS) is a chronic inflammatory neurodegenerative disorder that typically affects young adults and is primarily characterized by demyelinating lesions in the central nervous system (CNS). According to the Revised McDonald Criteria, the clinical diagnosis of MS can be established based on a combination of clinical observations, the presence of focal lesions in at least two distinct CNS areas on magnetic resonance imaging (MRI) and the detection of specific oligoclonal bands in the cerebrospinal fluid. Conventional MRI remains a cornerstone of MS diagnosis and disease monitoring, providing high-resolution assessments of lesion burden and brain atrophy. In addition, advanced MRI methods are increasingly applied in research settings to probe myelin integrity, iron deposition, and biochemical changes, with the potential to complement established diagnostic workflows in the future. Despite remarkable advances in the management of MS over the past two decades, complex differential diagnoses and the lack of effective imaging tools for therapy monitoring remain major obstacles, thus channeling the development of innovative molecular imaging probes that can be harnessed in clinical practice. Indeed, positron emission tomography (PET) has a significant potential to advance the contemporary diagnosis and management of MS. Given the solid body of evidence implicating myelin dysfunction in the pathophysiology of MS, myelin-targeted imaging probes have been developed, and are currently under clinical evaluation for MS diagnosis and therapy monitoring. In parallel, ligands for the 18 kDa translocator protein (TSPO) and the cannabinoid receptor type 2 (CB2R) have been employed to capture neuroinflammatory processes by visualizing microglial activation, while other tracers allow the assessment of synaptic integrity across various disease stages of MS. Further, PET probes have been employed to delineate the role of activated microglia and facilitate the assessment of synaptic dysfunction across all disease stages of MS. This review discusses the challenges and opportunities of translational molecular imaging by highlighting key molecular concepts that are currently leveraged for diagnostic imaging, patient stratification, therapy monitoring and drug development in MS. Moreover, we shed light on potential future developments that hold promise to advance our understanding of MS pathophysiology, with the ultimate goal to provide the best possible patient care for every individual MS patient.

## Introduction

**Figure 1:**
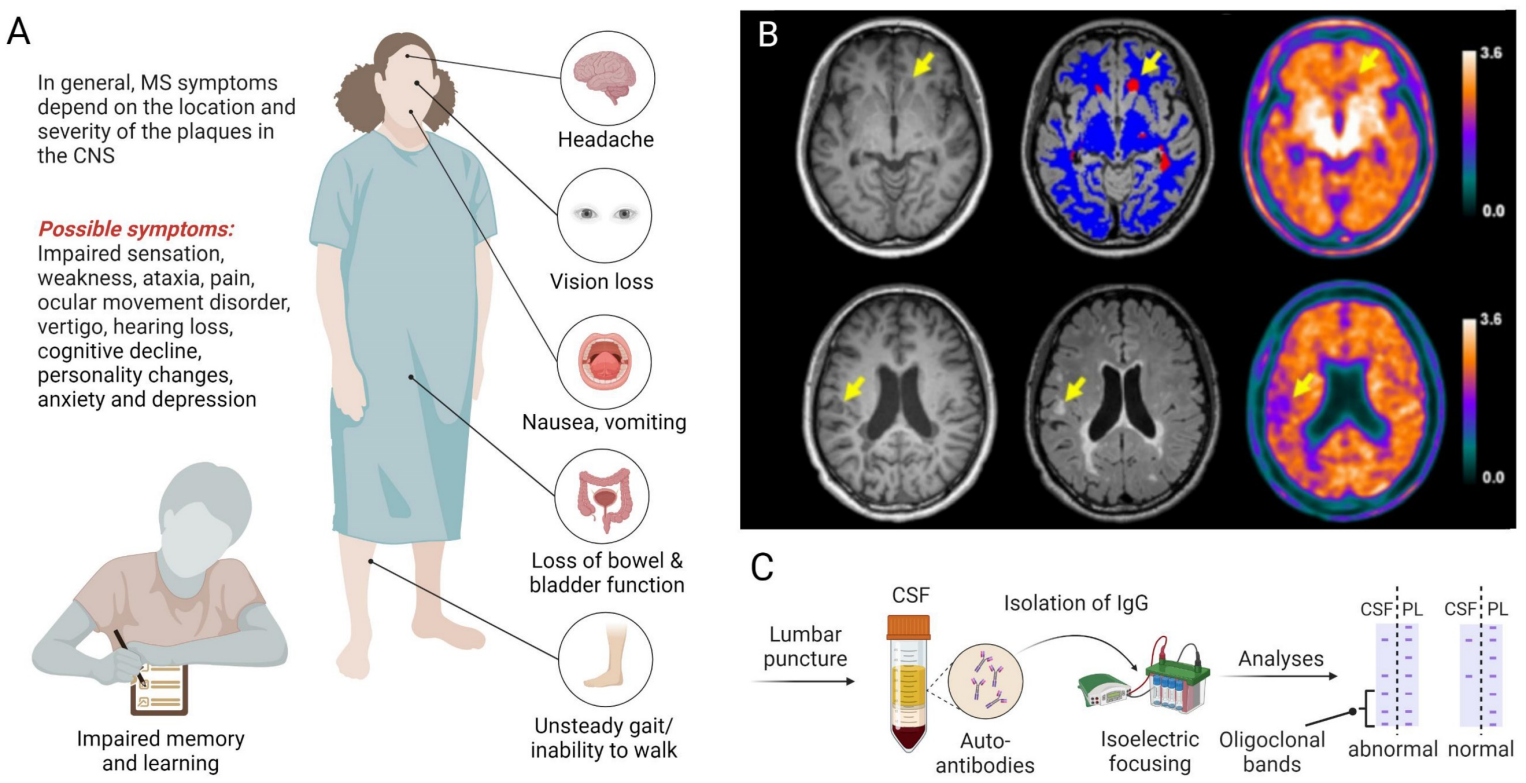
** Clinical presentation and biomarker-guided diagnosis of multiple sclerosis (MS). A.** The clinical manifestation of MS depends on the location and severity of lesions, involving a myriad of possible symptoms that affect several organs and ultimately lead to disability. **B.** Diagnostic imaging with T1-weighted (left panel), T2-weighted (middle panel) MRI and 18F-florbetapir PET (right panel) can be used to detect damaged white matter lesions (yellow arrows). Upper and lower rows represent examples from two distinct patients with relapsing-remitting MS according to the revised McDonalds criteria 10. Figure 1B, Adapted with permission from Elsevier, Zhang et al., doi: 10.1016/j.eclinm.2021.100982, copyright 2021. **C.** The diagnosis of MS is supported by laboratory findings, including the presence of specific oligoclonal bands in the CSF following lumbar puncture. *Abbreviations: CSF, cerebrospinal fluid; IgG, immunoglobulin G; MRI, magnetic resonance imaging; PET, positron emission tomography; PL, plasma.*

Multiple sclerosis (MS) is an inflammatory neurodegenerative disease that primarily affects the central nervous system (CNS) and is characterized by demyelinating lesions, gliosis and neuroaxonal degeneration, ultimately resulting in physical disability 1, 2. Notably, MS affects young adults - with a disease onset between 20-40 years of age - initially manifesting as reversible episodes of neurological deficits (relapsing-remitting MS) in most patients 3. Over the course of disease, however, patients are increasingly plagued by the development of persisting neurological deficits and permanent disability, a disease stage that is referred to as secondary progressive MS 4. Notably, around 10% of patients present with a progressive disease course from the onset and fall into the category of primary progressive MS. While geographical differences in the relative prevalence of MS subtypes were not significant 5, several studies have corroborated a higher prevalence of MS in women 6-8. Depending on the localization and severity of CNS lesions, clinical symptoms can include blurred vision with associated pain, impaired sensation in torso and extremities, weakness, ataxia, focal sensory disturbance, ocular movement dysfunction, vertigo, hearing loss and facial sensory disturbance (**Figure 1A**) 9. Given the broad range of possible symptoms, the diagnosis of MS relies on a combination of clinical observations, spatiotemporal dissemination of focal lesions within the CNS on neuroimaging (**Figure 1B**) and laboratory findings, including the presence of specific oligoclonal bands in the cerebrospinal fluid (**Figure 1C**), as outlined in the Revised McDonald Criteria 10.

**Figure 2:**
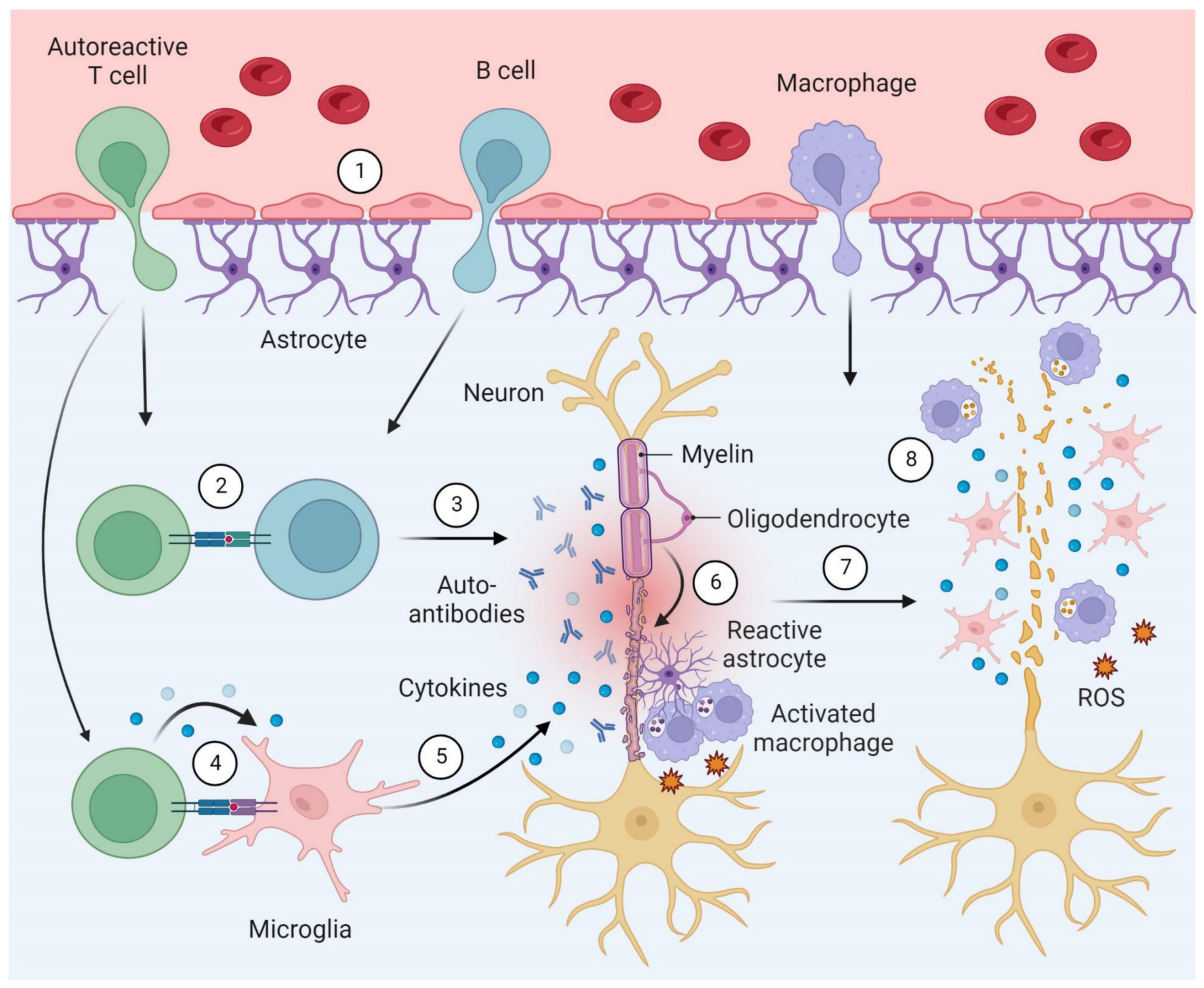
** Simplified model of the pathophysiology in multiple sclerosis (MS).** Pathophysiological hallmarks include (1) an impaired blood-brain barrier function, leading to infiltration of the central nervous system (CNS) by lymphocytes and macrophages. (2) Crosstalk between T and B lymphocytes prompts (3) the production of antibodies against oligodendrocyte antigens. (4) T cells interact with microglia, prompting (5) microglial activation and cytokine release. (6) Chronic inflammation ultimately leads to (6) axonal demyelination and the formation of glial scars by reactive astrocytes that are believed to exert protective functions by isolating damaged tissue. Macrophages are recruited to MS lesions, contributing to the phagocytosis of myelin debris - a process that triggers the release of pro-inflammatory cytokines and reactive oxygen species (ROS). (7) Axonal degeneration in MS lesions is driven by (8) activated microglial cells and macrophages.

Neuroinflammation plays a fundamental role at all stages of MS, where the innate and adaptive immune system are both implicated in the pathophysiology 2. Key hallmarks include CNS infiltration by macrophages and autoreactive T cells, facilitated by a leaky blood-brain barrier, as well as the production of antibodies against autoantigens. As the disease progresses, an excess release of pro-inflammatory mediators by activated microglia and astrocytes is observed, along with increasing numbers of autoantibody producing plasma cells (**Figure 2**) 3. Notably, these processes involve pivotal bidirectional cell-to-cell interactions. Indeed, cell-to-cell interactions between T cells and microglia have been a topic of extensive research in recent years. The current body of evidence suggests that T cells play a crucial role in the activation of resident microglia, leading to the release of pro-inflammatory cytokines and the destruction of myelin in MS (**Figure 2**) 11-14. Similarly, cell-to-cell interactions between T cells and B cells contribute to B cell differentiation, promoting the production of autoantibodies 11-13. Collectively, these processes ultimately result in axonal demyelination and degeneration 2.

To date, the treatment of MS can be divided into three main classes; 1) Short-term symptomatic therapies such as with nabiximols 15, 16 and fampridine 17, 18 to combat fatigue, pain and spasticity, 2) the management of acute MS relapses with high-dose corticosteroids, and 3) disease-modifying therapies (DMTs) that aim at attenuating the long-term clinical consequences of chronic inflammatory disease by attenuating peripheral immune cell activation and CNS infiltration 2, 3. Strenuous efforts have been made to develop effective DMTs in the past two decades, which has led to the approval of various effective DMTs to date. These drugs provide a versatility of options to tailor MS therapy to individual patient's needs. For instance, traditional injectable DMTs (e.g. interferon β or glatiramer acetate) have traditionally been considered the first-line treatment option due to their favorable risk-benefit profiles. However, traditional injectable DMTs are linked to injection-related adverse effects - as opposed to oral DMTs such as fingolimod, teriflunomide and dimethyl fumarate - and elicit only limited efficacy in a significant subpopulation of MS patients, often triggering a switch to more potent DMTs 19, 20. The concept of escalation therapy has proven particularly useful in clinical routine. The latter is based on an initial use of well-tolerated but moderately effective DMTs, followed by a switch to long-lasting immunosuppressive DMTs such as alemtuzumab or ocrelizumab along the disease course 21. In patients with aggressive disease or indicators of poor prognosis, an alternative therapeutic strategy known as induction therapy has been suggested, in which potent DMTs are used at disease onset to preclude the accumulation of irreversible neuronal damage 3. Regardless of the therapeutic strategy, there is a consensus that DMTs should be initiated as early as possible once the diagnosis of MS has been established. Despite substantial advances in the management of MS, differential diagnoses remain a challenge in contemporary clinical practice, at least in part, due to the broad range of diseases that mimic the clinical and radiological features of MS 22, 23. Improved biomarkers that facilitate the early assessment of therapy response and progression of disease are urgently needed. In addition to routinely used magnetic resonance imaging (MRI), molecular imaging modalities such as positron emission tomography (PET) and optical coherence tomography (OCT) are emerging as valuable tools with potential to improve our understanding of MS pathophysiology and support patient management. Indeed, OCT of the retinal nerve fiber layer (RNFL) has advanced rapidly and currently allows the assessment of neurodegeneration in MS patients with no clinical history of optic neuritis 24. While the annual thinning of RNFL is around 0.2 μm per year in healthy individuals, it can be up to ten times higher in MS patients 25, 26. Nonetheless, a recent prospective multicenter study concluded that there was no correlation of OCT data with disability progression in patients with relapsing-remitting MS 27. Additional studies are required to determine the diagnostic and prognostic value of OCT in relapsing-remitting MS.

While structural MRI is well established in the diagnosis and longitudinal monitoring of MS, its limited sensitivity for detecting early or diffuse neuroinflammatory changes, microglial activation, or evolving tissue damage in normal-appearing brain regions constrains its ability to fully capture disease activity and progression 28, 29. Advanced techniques such as magnetization transfer imaging (MTI), diffusion tensor imaging (DTI), and magnetic resonance spectroscopy (MRS) provide additional insights into myelin integrity, axonal damage, and metabolic alterations, but they are limited by modest specificity, technical variability, and lack of standardization across centers. As a result, these approaches remain largely confined to research rather than clinical practice. These gaps highlight the need for complementary molecular imaging tools that can directly target disease-relevant biological processes and provide quantitative insights into MS pathophysiology. Applications of translational molecular imaging with PET have proven useful for the non-invasive quantification of biological processes in preclinical and clinical research, including the assessment of myelin homeostasis, neuroinflammation and neurodegeneration 30-36. The principle of PET is based on the notion that PET radionuclides undergo radioactive decay by emitting a positron, which travels a certain distance (positron range) before it annihilates with an electron, generating a pair of 511 keV γ-rays that are concurrently released in opposite directions and can be captured by co-incidence detectors 37, 38. Substantial efforts have been made to develop PET probes that would facilitate drug development by enabling the *in vivo* assessment of protein expression and drug occupancy for target validation, drug biodistribution and pharmacokinetics experiments, as well as target engagement and micro-dosing studies 30, 37-39. These efforts have ultimately led to an arsenal of imaging tools to facilitate biomedical research from bench-to-bedside. PET harbors potential to monitor the *in vivo* efficacy of potential MS drug agents in the pipeline 34. Indeed, by providing real-time information on biochemical processes in living patients, PET probes have been employed to study MS and were established as clinical tools that provide information on MS onset and progression 32. Another critical advantage of PET imaging-guided drug development constitutes the capability to provide effective patient stratification during clinical MS trials. Despite the significant advances in MS-related imaging, the vast potential of PET is yet to be further elucidated and myelin-based imaging in MS patients has the potential to provide fundamental advances in the field. 40-42. While MRI has traditionally been employed to support research into the pathophysiology of myelin, as well as to facilitate the diagnostic workup for MS patients, MRI is primarily suited to detect alterations in physiological and anatomical properties, which are typically preceded by molecular and biochemical changes. In contrast, molecular imaging with PET enables identification and quantification of biochemical processes that precede anatomical changes, thereby identifying key determinants that can be leveraged for diagnostic and therapeutic purposes. Despite its molecular precision, PET imaging remains constrained by radiation exposure, high costs, and the need for radiopharmaceutical infrastructure, which limit its applicability in routine clinical MS care. MRI, in contrast, is widely accessible, non-invasive, and forms the basis of diagnostic criteria and prognostic value in MS 43. Nonetheless, PET imaging offers advantages in translational research by enabling high-sensitivity quantification of molecular targets and pharmacodynamic changes - parameters that are challenging to assess with conventional MRI techniques. As such, PET is best positioned as a complementary tool within the drug development continuum, particularly in early-phase clinical trials or mechanistic studies where target abundance and engagement, receptor occupancy, or *in vivo* biodistribution are critical endpoints. Moreover, post-mortem studies have significantly improved our understanding of molecular pathways that contribute to demyelination and axonal degeneration in MS. However, these studies are hampered by the lack of chronological information. Non-invasive *in vivo* imaging has the potential to overcome this limitation by providing a systematic reconstruction of the sequence of events in longitudinal studies, as well as by allowing for adequately powered clinical trials that encompass younger patients with prodromal and early stages of MS.

This work discusses the opportunities and limitations of available translational molecular imaging probes for MS, thereby focusing on molecular pathways that are harnessed to facilitate diagnostic imaging, patient stratification, therapy monitoring and drug development. Further, this review will provide a critical discussion of contemporary molecular concepts to visualize myelin dynamics, neuroinflammation and neurodegeneration, thereby exploring potential future trends in the field.

## Visualizing axonal myelin dynamics

**Figure 3:**
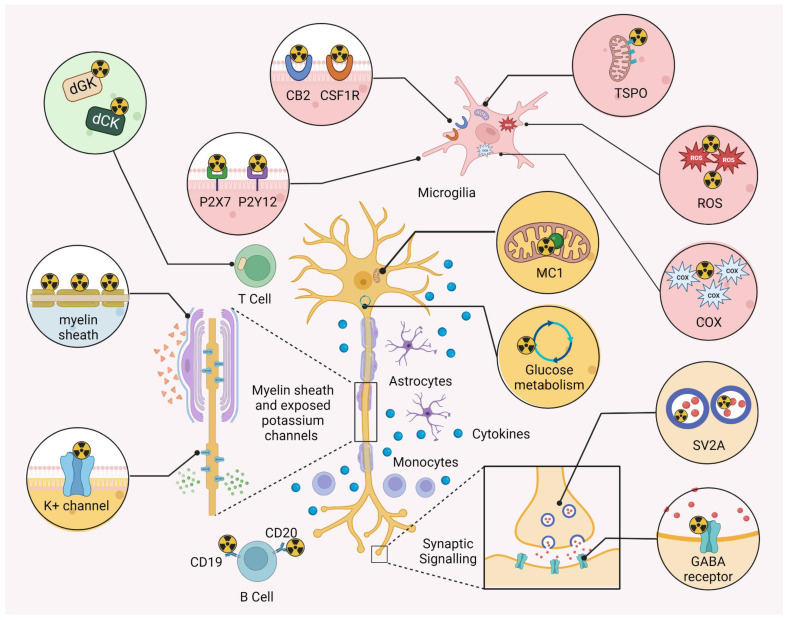
** Translational molecular imaging concepts in multiple sclerosis (MS).** Selected biological targets and processes that have been leveraged for positron emission tomography (PET) imaging in MS. PET can be used to improve diagnostic imaging and to facilitate drug discovery & development, and several probes have been successfully developed to non-invasively visualize myelination/demyelination by myelin-binding tracers and probes that image the potassium (K+) channel, which is exposed upon demyelination. Neuroinflammation imaging has traditionally relied on the visualization of biological targets that are overexpressed in activated microglia, including the 18-kDa translocator protein (TSPO), reactive oxygen species (ROS), cyclooxygenase (COX) enzymes, the cannabinoid type 2 receptor (CB2), colony stimulating factor 1 receptor (CSF1R) and purinergic receptors, P2X7 and P2Y12. Attempts to visualize the adaptive immune response have channeled the development of PET tracer that bind to CD19 and CD20 on B cells as well as probes that preferentially accumulate in T cells via a retention mechanism that involves binding to deoxyguanosine and deoxycytidine kinases (dGK and dCK). Neurodegeneration can be visualized by targeting glucose metabolism and mitochondrial complex I (MC-I), while synaptic integrity can be assessed with probes targeting the synaptic vesicle glycoprotein 2A (SV2A) or GABA receptors.

Myelin is characterized by a high lipid content, which changes the relaxation properties of adjacent water molecules, rendering MRI a suitable modality to detect advanced demyelinated lesions 44. Hence, MRI is typically used to support the diagnosis of MS in contemporary clinical practice 10. Despite the evident advantages, conventional MRI is limited due to its poor histopathological specificity. Indeed, while T1-weighted imaging shows some correlation with axonal density 45, it is difficult to discriminate between different processes such as demyelination, neuro-axonal damage, re-myelination and neuroinflammatory conditions 46. Similarly, it is difficult to distinguish between distinct states of MS plaques (e.g. a completely demyelinated, a demyelinated “shadow plaque” and a remyelinated lesion) 31. Along this line, the lack of histopathological specificity complicates the quantitative analysis of demyelination with MRI 31, 44. Quantitative MRI (qMRI) techniques such as MTI 47, 48 and DTI 49-51 are able to improve histopathological specificity to a limited extent, especially with the use of novel hardware for ultra-high gradient strength MRI. However, these qMRI techhniques are not currently used for myelin imaging in clinical routine. Accordingly, the non-invasive assessment of myelin dynamics constitutes an unmet medical need in contemporary management of MS and the development of improved non-invasive imaging tools are critical to improve patient care 52. There are three main classes of myelin-related radionuclide imaging; 1) Probes that accumulate in myelin-rich areas due to interactions with the β-sheet backbone in myelin basic protein (MBP), such as diamino stilbene (DAS) derivatives and probes that were initially developed for amyloid β (Aβ) imaging, 2) K+ channel-targeted tracers that specifically bind to K+ channels in exposed demyelinated axons and 3) coumarin-based probes that are believed to be capable of H-bonding with myelin. It should be noted, however, that the majority of reported myelin-targeted tracers primarily associate with myelin via hydrophobic interactions 53. An overview of the different classes of myelin PET ligands is provided in **Figure 3**.

### Targeting the β-sheet backbone in myelin

Despite the compelling rational of visualizing demyelinated lesions, a major challenge remains the presence of small areas of demyelination, which are typically masked due to the high abundance of surrounding intact myelin. In addition, since myelin is mostly composed of lipids, tracers for myelin are highly lipophilic, which typically results in high background radioactivity due to nonspecific tissue binding 44. Notwithstanding these challenges, significant efforts have resulted in the development of promising probes to visualize MS lesions by interacting with the β-sheet backbone in MBP. Most stilbene-based tracers share a common DAS pharmacophore, which facilitates interactions with MBP. It has been hypothesized that the planar structure of DAS favors interactions with the unique β-sheet assembly present in MBP 54, although the exact binding mode remains to be fully elucidated 55. The Congo red derivative, ^11^C-labeled 1, 4-bis (p-aminostyryl)-2-methoxybenzene (^11^C-BMB), was initially used for PET imaging of myelin 56, however, concerns have been raised about the histopathological specificity of ^11^C-BMB. The low histopathological specificity, along with a slow brain kinetics of the tracer and the short half-life of carbon-11 (20 min) have largely hampered the wider use of ^11^C-BMB. To overcome these limitations, a structurally optimized derivative of BMB, ^11^C-case imaging compound (^11^C-CIC) 57, was designed and synthesized based on an effective and more reliable radiolabeling procedure 57. In a first-in-human study, ^11^C-CIC was deemed sensitive in detecting differences of myelin density within MS lesions 58. Another lipophilic PET tracer, designated ^11^C-MeDAS (^11^C-*N-*methyl-4, 4′-diaminostilbene), was evaluated by small-animal PET in transgenic hyper-myelinated mice (Plp-AktDD) and respective control animals 59. ^11^C-MeDAS exhibited favorable pharmacokinetics, target binding and blood-brain barrier penetration 59. Demyelination and remyelination in the spinal cord of experimental autoimmune encephalitis (EAE) rat models of multiple sclerosis was successfully visualized by ^11^C-MeDAS PET 60, whereas the tracer demonstrated selectively for white matter regions in the brain and in spinal cord. Notably, ^11^C-MeDAS uptake was attenuated in the presence of inflammation-induced demyelination in EAE rats and progressively increased during the subsequent spontaneous remyelination, which was in concert with histopathological findings 54, 60. In recent years, significant research efforts have been devoted to validating the quantitative accuracy of ^11^C-MeDAS and identifying suitable radiofluorinated analogs with a longer physical half-life to allow their distribution to nuclear medicine facilities without an on-site cyclotron. Quantitative PET refers to the use of kinetic tracer modeling methods such as compartment modelling, Logan analysis 61 and parametric quantification in order to break down the PET signal into perfusion- and myelin-related tracer uptake 58, 62.

A recently reported first-in-human study assessing quantitative outcome measures demonstrated that ^11^C-MeDAS PET can be used for accurate quantification of myelin density in MS patients 58. The authors suggested that the reason for the improved efficacy of ^11^C-MeDAS over other lipophilic tracers is based upon its selectivity toward intact MBP. In particular, myelin damage was linked to a conformational change of MBP, resulting in the amelioration of the binding site for ^11^C-MeDAS 58.

Radiofluorinated derivatives of ^11^C-MeDAS, such as ^18^F-PENDAS 63, ^18^F-TAFDAS 64, or ^18^F-PEGMeDAS 65 were successfully synthesized and exhibited promising pharmacokinetic attributes by *in vivo* PET studies in rats. In conclusion, ^11^C-MeDAS and its fluorinated derivatives constitute promising stilbene-based tracers for the early delineation of MS lesions, with potential to facilitate diagnostic imaging in MS.

In a separate attempt to probe MBP, Aβ tracers - that emerged as invaluable tools to image amyloid deposition in neurodegenerative diseases such as Alzheimer's disease (AD) - have been repurposed for imaging white matter damage in MS 66, 67. These Aβ tracers bind to the white matter, independently of the presence or absence of Aβ deposition in the brain 68. Similar to the stilbene-based tracers, it is suggested that Aβ tracers bind to the white matter via hydrophobic interactions with MBP 69, although the detailed binding mechanism remains poorly understood. Notably, the β-sheet structure in both, amyloid peptide and MBP 70, 71, has been suggested as a common target for amyloid PET tracers 72. Stankoff et al. 73 was the first to propose a potential utility of the Aβ PET ligand ^11^C-Pittsburgh compound (^11^C-PiB) for myelin imaging. ^11^C-PiB has been widely used and validated for Aβ plaques imaging in AD, However, given that the tracer also exhibited excess binding in white matter regions, independent of the presence of Aβ plaques 74, the binding observed in the grey matter was attributed to interactions with myelin 73. A recent study demonstrated that ^11^C-PiB-based imaging was an effective tool for detecting demyelination in a non-human primate model of progressive MS 75. This data provided initial evidence for the utility of ^11^C-PiB as a diagnostic marker in progressive MS. In recent years, much work has been done on advancing the quantitative use of ^11^C-PiB in MS 41, 42. As such, quantitative studies revealed that ^11^C-PiB accumulation was up to 23% higher in myelin-rich white matter compared with grey matter regions 42. While parametric maps were significantly correlated with the expression of genes encoding for abundant proteins in the myelin sheath, no correlation was found between quantitative ^11^C-PiB PET and non-myelin-associated genes 42. Similarly, quantitative PET was employed to corroborate that ^11^C-PiB selectively binds to myelin in the white matter 41, including in the normal-appearing white matter 76. In a recent study investigating the regional distribution of myelin repair in relapsing-remitting MS patients, a selective failure of remyelination in periventricular white matter lesions was detected, indicating that lesion proximity to ventricles is associated with a failure of myelin repair 77. Notwithstanding the wide application and practical value, a key limitation of ^11^C-PiB PET is related to the sensitivity toward lysophosphatidylcholine (LPC) side effects - which may in some cases constitute a potential signal confounder 78. Further, the lack of opportunities for satellite distribution due to the short half-life of carbon-11 confines the use of this probe to nuclear medicine facilities with an on-site cyclotron. These drawbacks have prompted calls for the use of suitable radiofluorinated amyloid tracer analogs.

Radiofluorinated amyloid PET ligands such as ^18^F-florbetapir and ^18^F-florbetaben, have recently been tested in patients with MS 79, 80. In a study by Matías-Guiu et al. 79, twelve patients with established MS diagnosis and three healthy controls underwent MRI and ^18^F-florbetaben PET. ^18^F-Florbetaben uptake was measured in demyelinated plaques, normal-appearing white matter 80, and the grey matter 79. Mean SUV relative to cerebellum was higher in normal-appearing white matter than in damaged WM and was significantly correlated with severity of patient disability 79. Moreover, the latter study included a heterogeneous group of MS patients, including five individuals each with relapsing-remitting or secondary progressive MS, and two individuals with primary progressive MS. The authors found the most pronounced reduction of tracer uptake in the damaged white matter of individuals with relapsing-remitting MS 79. Similarly, a study with ^18^F-florbetapir that encompassed twelve MS patients found a significantly reduced tracer uptake in damaged white matter lesions compared to intact white matter areas for all patients included 80. The PET signal was significantly correlated with volumes of white matter regions on MRI, particularly in patients that were in the active phase of disease. These findings were further supported by the recent report of Zhang et al., in which the authors conducted a longitudinal study examining the utility of ^18^F-florbetapir for monitoring demyelination lesions 81. In the latter study, it was observed that ^18^F-florbetapir uptake successfully differentiated between demyelination and inflammatory edema, which is typically indistinguishable on MRI. Moreover, results of the Expanded Disability Status Scale (EDSS) 82, a widely used clinical metric to assess MS-related disability, was correlated with the ^18^F-florbetapir PET signal, thus indicating that ^18^F-florbetapir may constitute a suitable tool for disease monitoring in MS 83. In another ^18^F-florbetapir study encompassing 18 relapsing-remitting MS patients and 12 healthy controls, it was shown that demyelination in MS was not restricted to lesions detected through conventional MRI, but also involved the normal appearing white matter 40. In addition to PET, SPECT probes have been suggested for myelin imaging in MS, with four diaryl oxadiazole derivatives that were evaluated as novel myelin-targeted probes 84. Among these compounds, ^123^I-1,3,4-PODP-DM-based SPECT/CT was used to visualize LPC-induced demyelination in the mouse brain. While these results indicated a potential utility of ^123^I-1,3,4-PODP-DM as a SPECT probe for myelin imaging in MS, the available data stems from preclinical observations and clinical data are required to corroborate the utility of this probe to monitor disease progression as well as to predict response to therapy.

While the conventional wisdom implies that Aβ tracers predominantly target myelin in MS patients, this concept has been increasingly challenged by the notion that Aβ itself may constitute a biomarker of demyelination 80. Indeed, various reports corroborated that the amyloid precursor protein (APP) accumulates in damaged axons 85-87, 88. In particular, a high APP immunoreactivity was observed in actively demyelinating MS lesions but not in chronic lesions, suggesting a peculiar alteration of APP abundancy across disease stages 89. Aβ may be involved in axonal remyelination, given that the β-site APP-cleaving enzyme 1 (BACE1) is involved in the cleavage of neuregulin 1, a protein that plays a critical role in oligodendrocyte differentiation and remyelination. Intriguingly, the genetic deletion of BACE1 during development leads to hypomyelination in mice 90. While contemporary evidence points towards a potential protective role of Aβ in MS, CSF Aβ_1-42_ has been suggested as a putative biomarker of MS progression 91. In conclusion, demyelinated lesions typically appear as areas of low tracer uptake on amyloid PET in MS patients. As such, contemporary evidence supports the concept that the attenuated ^18^F-florbetapir uptake in MS lesions is indeed driven by the reduced abundancy of myelin. Nonetheless, further studies are required to delineate the molecular mechanism by which amyloid PET ligands are capable of detecting demyelination. Despite the remaining knowledge gaps, commercial availability due to the widely established production sites for the use in AD, as well as encouraging preclinical and clinical MS studies to date render amyloid PET a valid option to be further elucidated for diagnostic applications in MS.

### Probing demyelination via exposed K+ channels

An important consideration for probes that identify demyelinated lesions via a “negative” signal, which is characterized by attenuated tracer accumulation in the lesion compared to adjacent areas, is that spillover effects from adjacent areas hamper the quantitative assessment of myelin abundancy. Accordingly, a different approach has been proposed that leverages PET tracer selectively targeted toward proteins that become more accessible in demyelinated axons (**Figure 3**), ultimately resulting in an excess tracer uptake at sites of demyelination (“positive” signal) 44. Upon demyelination, axonal K+ channels are upregulated and increasingly exposed to the surrounding microenvironment 92. Notably, it was found that 4-aminopyridine (fampridine) and other aminopyridines act as K+ channel blockers by forming several hydrogen bonds with the K+ channel α-subunit 93. Fampridine is the most extensively investigated drug in this class and is currently approved for symptomatic treatment for walking disability in patients with MS 94. Radiolabeled derivatives of fampridine were suggested as potential PET tracers for imaging demyelinated lesions. Indeed, this concept has been leveraged in experimental models, and its successful application in humans could provide transformative insights; not only could it become a powerful tool for characterizing the extent of demyelination in the brains of MS patients, but also serve as a marker for therapy monitoring. Further, combined use of fampridine-based PET with MRI provides unique insights by enabling simultaneous quantification of surviving demyelinated axons and their subsequent myelin repair. Such a hybrid imaging approach may lay the foundation for a powerful outcome measure in clinical trials assessing the utility of novel remyelination therapies 31. The development of fampridine analogues for PET imaging is presented as a case example in the drug development chapter to illustrate the role of molecular imaging in supporting lead identification and optimization during the drug development process for MS.

### Myelin imaging with coumarin-based probes

Coumarins belong to a family of heterocyclic benzopyrones that can be naturally derived from plants and exhibit a wide array of biological activities, providing a versatile therapeutic profile 95. Accordingly, coumarins have been harnessed as anticoagulant, antibacterial, anti-inflammatory, antioxidant, antitumor, antiviral and neuroprotective agents 96. For instance, 3-(4-aminophenyl)-coumarin has been suggested as an exploratory therapy for AD 97-100. Similarly, coumarin-based molecular imaging probes have been developed for fluorescence 101 and PET 102 imaging of myelin. Notably, coumarin-based probes are believed to exhibit superior binding properties toward myelin compared to stilbene-based tracers due to inherent structural characteristics, including the potential capability to undergo hydrogen binding 102. It was hypothesized that interactions between coumarins and myelin were likely to take place at myelin sites that are enriched with nonpolar amino acids and fatty acids of myelin-associated lipids. Given that structure-activity relationship studies with coumarin derivatives revealed that the lack of a dimethylamino group substantially reduced the affinity for MBP, it is hypothesized that coumarins gain access to myelin binding sites by specific interactions through their dimethylamino groups, 102. Further, studies unveiled that proteins in the myelin sheath have an overall positive charge at physiological pH due to an excess of lysine and arginine residues 103, potentially acting as hydrogen bond donors to the dimethylamino moiety in coumarins 102. Given the promising pharmacological properties, Watanabe et al. recently reported on the development of several radioiodine labeled coumarin derivatives, of which radioiodine-labeled target compound **21** 104 has been deemed potentially useful for myelin-targeted SPECT imaging in MS. In addition to the suggested interactions with myelin, recent molecular modelling studies have raised the possibility that some coumarin derivatives may act as K+ channel blockers 105. Such observations were made with several coumarin derivatives, including xanthotoxin 106, osthole 107, coumarsabin 108, and furochromones 109. Nonetheless, it should be noted that the development of coumarin-based tracers for myelin imaging is still in its infancy and more work remains to be done to assess their potential for clinical applications in MS and beyond.

### Comparison studies of myelin-targeted probes

To date, attempts to compare different classes of tracers for myelin imaging are limited. Nonetheless, a few comparison studies can be found in the literature. For instance, ^11^C-PiB uptake was compared with ^11^C-CIC and ^11^C-MeDAS in rodents 110. All tracers showed fast brain uptake and distribution volumes that were largely in accordance with myelin-rich regions, however, ^11^C-PiB revealed low uptake in some myelinated regions, including the cerebellum, midbrain and brain stem. Overall, ^11^C-MeDAS distribution seemed to correlate better with myelin density that the distributions of ^11^C-PiB and ^11^C-CIC, indicating that ^11^C-MeDAS was more suitable for quantitative myelin imaging 54, 110. Another comparison study of ^18^F-florbetaben, ^18^F-florbetapir, ^18^F-flutemetamol, ^11^C-MeDAS, and ^11^C-PiB in baboons concluded that ^18^F-florbetapir and ^18^F-florbetaben exhibited superior tracer performance characteristics and indicated that ^18^F-florbetapir and ^18^F-florbetaben constitute the most suitable probes for myelin imaging 111. Nonetheless, given the small number of comparison studies in this field, including the limited data in higher species, it seems premature to draw conclusions on the most suitable class of tracers for myelin imaging in patients. Hence, there is a need for more comparison studies where the efficiency and tracer performance characteristics are compared in non-human primates and humans.

## Informing drug development in multiple sclerosis

Contemporary drug development constitutes an exceptionally costly and complex endeavor, with a strikingly low overall success rate. Between 2009 and 2018, the median research and development (R&D) cost per FDA-approved drug was estimated at $985 million 112. Against this backdrop, translational molecular imaging has emerged as a valuable strategy to streamline drug development and enhance the probability of success. These efforts have led to the establishment of an extensive imaging toolkit that supports biomedical research from bench to bedside. Although the integration of PET imaging in drug development for MS is still in its early stages, a growing number of radioligands are currently being investigated in clinical trials (**Tables 1** and** 2**). In this section, we delineate how molecular imaging can inform critical stages of drug development in MS, drawing on selected case studies to illustrate its potential 38.

**Figure 4:**
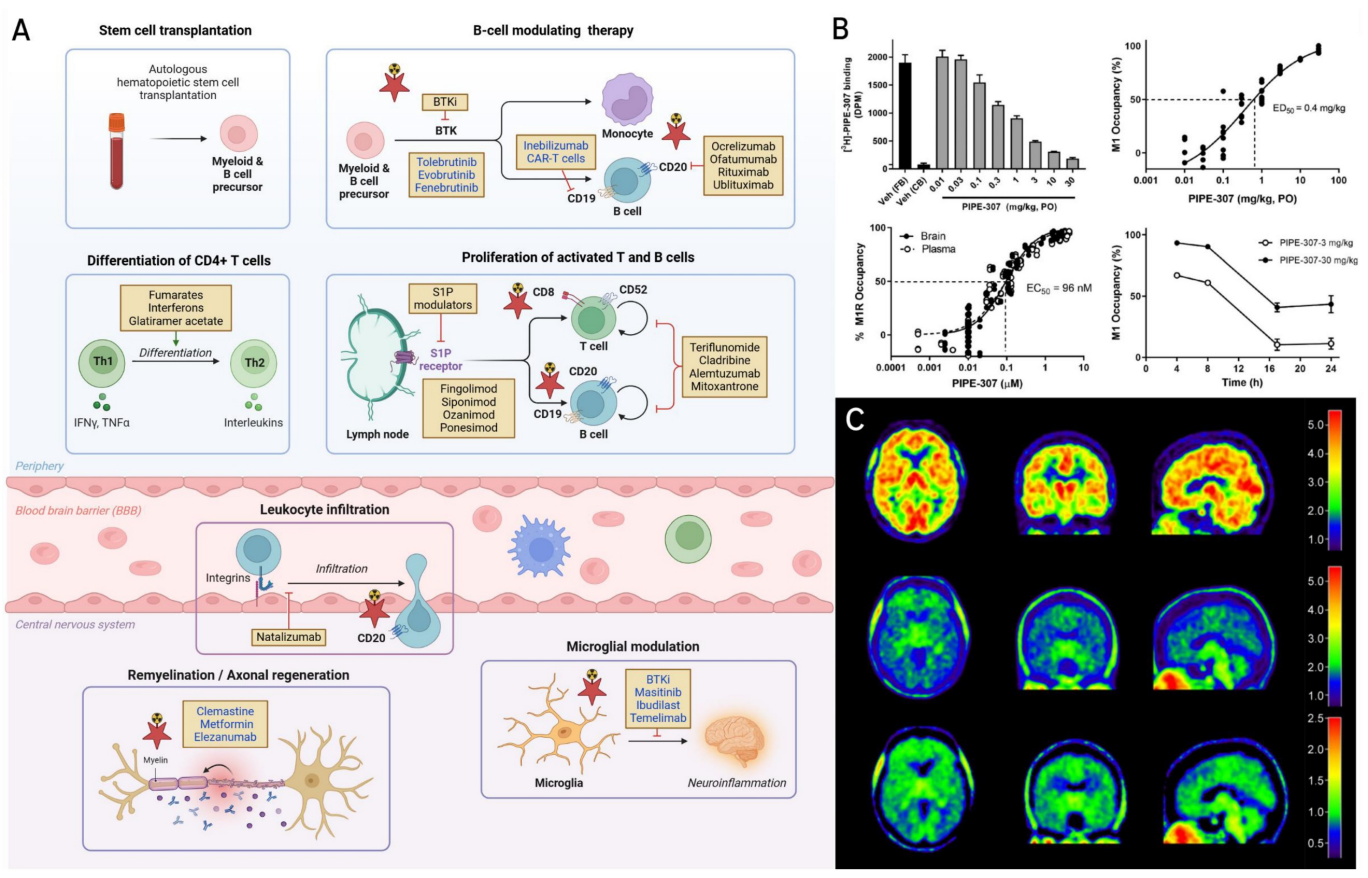
** Interplay between imaging and disease modifying therapies (DMTs) in multiple sclerosis (MS).** A. Mechanistic landscape of current and emerging (in blue colour) DMTs in MS. Primary sites of action of established and emerging DMTs (highlighted in blue) are shown across the MS disease continuum, spanning peripheral immune modulation, leukocyte trafficking and central nervous system (CNS) infiltration, microglial modulation, and remyelination therapy. B cell-depleting therapies include approved anti-CD20 monoclonal antibodies such as ocrelizumab, ofatumumab, rituximab, and ublituximab, as well as investigational anti-CD19 approaches (inebilizumab and CD19 chimeric antigen receptor T (CAR-T) cells). Bruton's tyrosine kinase (BTK) inhibitors such as tolebrutinib, evobrutinib, and fenebrutinib modulate signaling in B cells and myeloid lineage cells and are currently in late-stage development. T cell-modulating agents, including interferon-β (IFN-β), glatiramer acetate, fumarates (e.g. dimethyl fumarate), and teriflunomide, act by modulating the balance of pro- and anti-inflammatory T cell subsets, limiting T cell activation, and reducing the proliferation of lymphocytes. Sphingosine-1-phosphate (S1P) receptor modulators - including fingolimod, siponimod, ozanimod and ponesimod - block lymphocyte egress from secondary lymphoid tissues, thereby limiting CNS infiltration, while natalizumab prevents immune cell entry by inhibiting α4β1 integrin-mediated adhesion at the blood-brain barrier (BBB). DMTs such as cladribine, alemtuzumab, and mitoxantrone exert broader cytotoxic effects on proliferating immune cells. Within the CNS, masitinib and ibudilast inhibit the activation of microglia and mast cells, while temelimab, a monoclonal antibody targeting the envelope protein of the human endogenous retrovirus-W (HERV-W ENV), reduces innate immune activation and hold potential to preserve myelin integrity. Clemastine fumarate, a muscarinic receptor antagonist, enhances remyelination by promoting differentiation of oligodendrocyte precursor cells (OPCs), while metformin restores the regenerative capacity of aged OPCs. Elezanumab, an anti-repulsive guidance molecule-a (anti-RGMa) antibody, facilitates axonal regeneration and remyelination, and BTK inhibitors may additionally modulate CNS-resident microglia and macrophages. Pathways that can be probed with molecular imaging are denoted by a red star. Collectively, the therapeutic landscape of MS is rapidly evolving toward CNS-penetrant, pathway-specific agents that not only suppress peripheral inflammation but also directly modulate CNS disease processes and promote tissue repair. B. Receptor occupancy of PIPE-307. PIPE-307 displays dose-dependent M1 receptor occupancy in mouse brain following oral administration, with an ED₅₀ of 0.4 mg/kg. Total and nonspecific binding were determined using 3H-PIPE-307 in forebrain and cerebellum, respectively. Time course studies indicate that 30 mg/kg achieves rapid, full occupancy with ~50% occupancy persisting at 16 h, while 3 mg/kg maintains ~50% occupancy for approximately 10 h. This study exemplifies how tritiated radioligands can be used to quantify *ex vivo* target engagement and inform dose selection during early drug development. *Figure. 4B was reprinted with permission from National Academy of Sciences, Poon et al. copyright (2024) doi: 10.1073/pnas.2407974121.* C. Brain distribution of TSPO ligand 18F-DPA-714. Following intravenous administration of 304 MBq 18F-DPA-714, brain radioactivity was visualized at early (0-10 min) and late (30-90 min) post-injection time points. Parametric distribution volume ratio (DVR) maps were generated using the cerebellum as a reference region. This study illustrates the use of PET imaging to quantify TSPO expression as a surrogate for microglial activation in humans. Red star highlights imaging targets leveraged in MS drug development. *Figure. 4B was reprinted with permission from Elsevier, Arlicot et al. copyright (2012) doi: 10.1016/j.nucmedbio.2011.10.012.*

The past two decades have witnessed a remarkable expansion in the therapeutic strategies available for the treatment of MS, shifting the landscape from nonspecific immunomodulation toward more targeted strategies that suppress peripheral inflammation and increasingly targeting CNS pathology 113-115. Disease-modifying therapies (DMTs) constitute the backbone of MS treatment and act at various stages of the immunopathological cascade, including immune cell differentiation, trafficking across the blood-brain barrier (BBB), microglial activation, and myelin repair (**Figure 4A**). First-generation DMTs such as interferon-β and glatiramer acetate remain widely used, particularly in patients with milder relapsing forms of MS, where they modulate T cell polarization and suppress antigen presentation by peripheral immune cells. These agents have been complemented by oral DMTs such as fumarates, which exert immunomodulatory and cytoprotective effects, at least in part, through activation of the nuclear factor erythroid 2-related factor 2 (Nrf2) pathway 116, and teriflunomide, which inhibits pyrimidine biosynthesis and reduces lymphocyte proliferation 117. A significant advance in MS therapy has been the development of monoclonal antibodies targeting CD20 expressed on B cells 118. Of note, ocrelizumab, ofatumumab, rituximab, and ublituximab mediate B cell depletion via antibody- or complement-dependent cytotoxicity, showing robust efficacy in reducing relapse rates, lesion activity, and disability progression in both relapsing-remitting and primary progressive MS 115. Investigational anti-CD19 agents such as inebilizumab and CD19-targeted chimeric antigen receptor T (CAR-T) cells hold potential to extend the therapeutic reach by targeting earlier B cell lineages, as CD19 was shown to be abundantly expressed at early development stages of B cells 119. Other established therapies include sphingosine-1-phosphate (S1P) receptor modulators, including fingolimod, siponimod, ozanimod, and ponesimod, prevent lymphocyte egress from lymphoid tissues, thereby limiting CNS infiltration 120, 121.

Natalizumab, a monoclonal antibody targeting the α4β1 integrin, blocks leukocyte adhesion and transmigration across the BBB and is associated with high efficacy in relapsing forms of MS, albeit with a suggested risk of progressive multifocal leukoencephalopathy in susceptible individuals 122, 123. Beyond immune suppression, emerging therapies are being designed to address inflammation localized within the CNS and promote repair. Bruton's tyrosine kinase (BTK) inhibitors, including tolebrutinib, evobrutinib, and fenebrutinib, modulate B cell signaling and innate immune responses mediated by microglia 124, 125. These agents are currently in late-phase development and offer the potential to cross the BBB and target CNS resident microglia 126-129. In addition, strategies focused on neuroprotection and remyelination are gaining momentum. Masitinib, a tyrosine kinase inhibitor, and ibudilast, a phosphodiesterase inhibitor, have shown promise in reducing microglial activation and oxidative damage in progressive MS 130, 131. Temelimab, a monoclonal antibody against the envelope protein of the human endogenous retrovirus-W (HERV-W ENV), may counteract microglia-mediated demyelination and support myelin preservation 132. Several remyelination therapies are also under investigation. Clemastine fumarate, a muscarinic receptor antagonist, enhances oligodendrocyte precursor cell (OPC) differentiation and was among the first agents to demonstrate functional remyelination in a clinical trial 133. Similarly, metformin has been shown to rejuvenate aged OPCs and restore their remyelination potential 134.

Finally, the anti-repulsive guidance molecule A (RGMa) antibody, elezanumab, facilitates axonal regeneration and remyelination and is currently being explored in relapsing forms of MS (NCT03737851) 135. Collectively, the therapeutic pipeline is rapidly evolving beyond conventional immune modulation, toward mechanistically targeted, CNS-penetrant interventions that may ultimately transform treatment paradigms in MS. Several of the pathways modulated by DMTs can also be visualized with molecular imaging tools, creating opportunities to non-invasively monitor treatment effects and guide therapeutic decision-making. These intersections between imaging and pharmacotherapy are illustrated in **Figure 4A**, with pathways highlighted that are currently accessible by PET.

The integration of molecular imaging into drug development pipelines has opened new avenues to accelerate therapeutic innovation and refine clinical decision-making. While the diagnostic utility of PET lies in its capacity to detect and characterize disease-related processes, its value in drug development is centered around enabling *in vivo* assessments of drug biodistribution and pharmacokinetics, as well as in assessing pharmacodynamic response markers. Within the realm of MS, PET imaging serves as a transformative tool to non-invasively quantify target occupancy and monitor treatment-induced changes in neuroinflammation, demyelination, and neurodegeneration across different species 32. Notably, such assessments provide key pharmacological insights, including central nervous system penetration, target specificity and selectivity, as well as the relationship between exposure in the target compartment and biological effect. A notable example is the use of ^11^C-PBR28 PET to quantify neuroinflammation by targeting the 18-kDa translocator protein (TSPO), which is upregulated on activated microglia. Indeed, studies have demonstrated significantly elevated TSPO levels in MS patients compared to healthy controls, underscoring the utility of TSPO PET as a biomarker of disease activity and a potential endpoint for therapeutic trials 136. Collectively, PET imaging offers a powerful platform to de-risk early-stage drug development, enhance pharmacological precision, and support adaptive trial designs. As molecular imaging tools continue to evolve, their application in MS drug development is expected to expand - facilitating the emergence of tailored therapeutic strategies that address the complex pathophysiology of the disease while maximizing clinical benefit. In this chapter, we illustrate the interplay between molecular imaging and therapeutic development through selected case examples, spanning the continuum from target identification and validation to lead optimization, entry to human (EIH) studies and subsequent clinical development.

### Target identification and validation

The target identification and validation stages focus on establishing the presence of a molecular target under pathophysiological conditions and verifying that its modulation may confer therapeutic benefit. Translational PET imaging enables non-invasive, quantitative validation of target expression in living subjects, providing direct evidence that a target is accessible and pharmacologically tractable in the human CNS. Of note, while traditional approaches to interrogating neurodegeneration or neuroimmune pathology in MS have largely relied on post-mortem analyses, these methods preclude longitudinal assessments in living patients and are limited by the instability of target proteins *ex vivo* - hence they are prone to bias that results from the post-mortem delay prior to tissue fixation. In contrast, non-invasive PET imaging provides an effective opportunity to capture dynamic biological processes *in vivo*. Two prominent strategies under evaluation are synaptic density imaging with synaptic vesicle glycoprotein 2A (SV2A)-targeted PET and immuno-PET approaches for visualizing immune cell populations.

The extent to which synaptic degeneration contributes to MS pathophysiology remains a subject of debate, in part due to conflicting post-mortem findings. Indeed, a systematic review by Mock et al. concluded that the link between synaptic loss and MS is still controversial, highlighting the need for *in vivo* techniques that circumvent the limitations of post-mortem tissue analyses 137. Quantitative PET imaging offers a solution by allowing for the early live detection of synaptic dysfunction - before structural abnormalities become apparent on MRI 138. SV2A is ubiquitously expressed in presynaptic terminals and serves as a robust biomarker of synaptic density. Several PET tracers have been developed for this purpose, including ligands from the UCB- and SDM-series. Notably, ^11^C-UCB-J 139 and ^18^F-labeled derivatives such as ^18^F-SDM-7 and ^18^F-SDM-8 140, 141 have demonstrated high selectivity and brain penetration, with successful clinical application in neurodegenerative diseases such as PD, where significantly reduced SV2A binding was observed in the substantia nigra 142. A clinical study assessing the utility of ^18^F-SDM-8 in a cohort of 30 MS patients (NCT04634994) has been initiated, and its results could shed light on the utility of SV2A PET as a biomarker for synaptic pathology in MS. Beyond neurodegeneration, molecular imaging can be leveraged for target validation by visualizing components of the adaptive immune response. Along this line, the capability to assess the involvement of B and T lymphocytes would be of crucial interest, given the body of evidence supporting the concept of autoimmune-like activity against CNS autoantigens in MS 1. The breakdown of tolerance to autoantigens activates previously dormant autoreactive B and T cells in MS patients, although the underlying triggers are controversially discussed 143. The availability of non-invasive imaging tools to visualize CNS infiltration by specific lymphocytes would substantially facilitate research into the origins of autoantigen generation, potentially opening up new avenues for therapeutic intervention. While activated B cells were visualized by targeting CD19 or CD20 with PET using ^64^Cu-CD19-mAb 144 or ^64^Cu-rituximab 145, respectively, activated T cells have been investigated in MS using a tracer known as ^18^F-F-AraG 146, which is a substrate of abundantly present kinases in T cells. Similarly, immuno-PET with ^89^Zr-labelled antibodies is increasingly popular due to the long half-life of ^89^Zr (t_1/2_ = 3.3 days) 147, as evidenced by its use in several existing clinical trials (**Tables 1** and** 2**). For instance, ^89^Zr-Df-crefmirlimab, which shows strong affinity to CD8+ T-cells, has been used in clinical trials to identify the distribution of CD8+ T cells in the CNS of adults with MS and hence provide in-vivo imaging of the immune response, thereby enabling *in vivo* imaging of the adaptive immune response (NCT05849467). Notably, the ability to longitudinally track immune cell dynamics could be harnessed to identify and validate novel targets in MS. By capturing temporal shifts in B or T cell activity following intervention, immuno-PET offers a promising strategy to assess pharmacodynamic effects and establish the translational validity of a given therapeutic pathway.

### Lead identification and optimization

Lead identification involves screening candidate molecules for their ability to modulate the intended biological target, followed by structure-activity relationship studies and chemical optimization. PET imaging plays a dual role: first, in the development of selective radiotracers as tool compounds to support lead selection and optimization via assessment of drug-target interactions, and second, by probing the pharmacodynamic effects of advanced molecules in preclinical animal models that include not only rodents but also non-human primates. When a lead structure is already available, the structural scaffold may serve as a basis to develop a suitable PET radioligand in parallel to the actual drug development efforts. As discussed before, PET tracers derived from fampridine for targeting demyelinated axons via exposed potassium channels constitutes a notable example in this regard. Potassium channel blockers represent a class of neuroprotective agents that mitigate the functional consequences of demyelination by inhibiting the pathological efflux of K⁺ ions through voltage-gated potassium (Kv) channels exposed on denuded axons (**Figure 3**) 148. The molecular mechanism of fampridine action - through blockade of Kv channels via hydrogen bonding with the channel's α-subunit - was first elucidated through electrophysiological studies and later substantiated by molecular docking analyses 93, 149. Despite its clinical success, fampridine exhibits dose-dependent off-target activity and lacks subtype-selectivity for Kv channels 150. This underscores the need for more selective Kv channel modulators that preserve the therapeutic benefit while minimizing unwanted effects. Inspired by the story of fampridine, a series of structurally related analogues have been developed for PET imaging to visualize Kv channel exposure in demyelinated axons. These include ^11^C-3-methyl-4-aminopyridine (^11^C-3Me4AP) 151, ^18^F-3-fluoro-4-aminopyridine (^18^F-3F4AP) 152, and ^11^C-3-methoxy-4-aminopyridine (^11^C-3MeO4AP) 153. Among these, ^18^F-3F4AP and ^11^C-3MeO4AP have shown favorable brain penetration, metabolic stability, and sensitivity to focal demyelination in non-human primates 152, 153. Preliminary first-in-human images of ^18^F-3F4AP have recently been disclosed, and two clinical trials are underway to evaluate this tracer in patients with MS (NCT04699747) and other demyelinating disorders (NCT04710550) 154, 155. If validated, ^18^F-3F4AP PET imaging could represent a paradigm shift in the non-invasive assessment of demyelination, providing spatially resolved, quantitative measures to inform both diagnostic and therapeutic strategies. Following clinical validation, these probes could serve as valuable tools to support the optimization of next-generation Kv channel blockers and accelerate their progression toward human translation.

### Human translation and clinical development

Early clinical development focuses on demonstrating a suitable pharmacokinetic profile and pharmacological activity at safe doses in humans, thereby establishing a therapeutic window. A major advantage of molecular imaging lies in its versatility, allowing both purely diagnostic imaging for patient stratification as well as target occupancy and therapy monitoring to optimize the dosing regimen in patients. A prominent example of molecular imaging facilitating clinical development in MS is PIPE-307, a selective M1 receptor antagonist designed to promote remyelination, which was supported by PET-based receptor occupancy studies in the clinic. Emerging evidence suggests that the differentiation of oligodendrocyte precursor cells (OPCs) is negatively regulated by the M1 subtype of the muscarinic acetylcholine receptor - with studies demonstrating that pharmacological blockade or genetic deletion of M1 receptor promotes oligodendrocyte maturation and enhances remyelination in preclinical models 156-158. Building on these mechanistic insights, a high-throughput screening campaign was conducted to identify small-molecule M1R antagonists using a novel combination of nanopillar array platforms and fluorescence-based readouts 157. Among the resulting candidate compounds, PIPE-307 emerged as a highly potent and selective M1R antagonist, exhibiting nanomolar affinity in both mouse and human brain tissue homogenates, as determined using tritiated PIPE-307 (^3^H-PIPE-307, **Figure 4B**) 159. Counter screening against other muscarinic receptor subtypes (M2-M5) revealed >10-fold functional selectivity, underscoring its utility for selective targeting of M1R. Of note, radiolabeling with tritium is a widely adopted strategy in the early stages of drug development, offering a minimally disruptive method to confirm target abundance and drug-target interactions *in vitro* or *ex vivo* 160. Once target affinity is established, PET radiolabeling with carbon-11 or fluorine-18 is typically pursued to enable quantitative imaging *in vivo*. In the case of PIPE-307, its favorable binding profile justified the development of the PET-compatible analogue, ^11^C-PIPE-307, which was subsequently evaluated in a human receptor occupancy study (NCT04941781) - with results currently pending. In parallel, PIPE-307 has advanced into exploratory clinical development with promising outcomes. Proof of concept with PIPE-307 was obtained in the EAE mouse model, where treatment resulted in robust axonal remyelination, consistent with its proposed mechanism of action 159. Notably, PIPE-307 has since successfully completed a Phase I clinical trial without evidence of cognitive adverse effects, and a Phase II trial (NCT06083753) is currently underway. The dual functionality of PIPE-307 - as both a therapeutic agent and a radiolabeled molecular imaging probe - exemplifies an effective paradigm shift in MS drug development that seamlessly integrates diagnostics and treatment efforts. Indeed, this approach could be extended to additional molecular targets. For instance, PIPE-791, a lysophosphatidic acid (LPA) receptor antagonist, has demonstrated the ability to modulate immunoinflammatory signaling and promote remyelination 161. Receptor occupancy was confirmed using a tritiated analogue (^3^H-PIPE-791), supporting the potential for a theranostic application. However, translational studies employing a PET-compatible radioligand are warranted to evaluate the clinical feasibility and added value of this strategy. Beyond the established targets discussed above, additional clinical imaging trials are expanding the translational toolkit for MS. For example, a study investigating the combined use of the nasal anti-CD3 agent foralumab with ^18^F-PBR06 PET 162 to assess the capacity of this class of antibodies to attenuate microglial activation (NCT06292923). Another innovative study aims to assess remyelination of the optic nerve using ^18^F-florbetaben PET in conjunction with the N-methyl-D-aspartate (NMDA) receptor antagonist, ifenprodil, marking an effort to evaluate therapeutic efficacy of GluN2B-subtype selective NMDA inhibitors in MS (NCT06330077). These trials exemplify the increasing scope of PET imaging not only for mechanism-of-action studies, but also for guiding therapeutic interventions and individualized care.

## Molecular imaging of neuroinflammation

For decades, TSPO-targeted PET has been the cornerstone of neuroinflammation imaging in the living human brain 32. While TSPO is expressed in the outer mitochondrial membrane, a solid body of evidence suggests that it is highly abundant on activated microglia, rendering TSPO-targeted probes suitable tools to assess neuroinflammation in various neurological disorder, including MS (**Figure 3**) 163.

A series of TSPO PET ligands were successfully translated to humans, including the first-generation PET tracer, *(R)-*^11^C-PK11195, and subsequently developed analogs with improved signal-to-noise ratio. The latter include, but are not limited to, ^11^C-DPA-713, ^18^F-DPA-714 (**Figure 4C**), ^11^C-PBR28, ^18^F-PBR111 and ^11^C-ER176 164. Many of these probes were leveraged to study neuroinflammation in MS, providing crucial insights into the role of the immune system in the pathophysiology of MS. For instance, TSPO PET unveiled that activated microglia were involved across all stages of MS - with PET signal intensities that were closely linked to the severity of clinical symptoms 165-169. Similarly, TSPO PET with ^11^C-PBR28 provided non-invasively quantification of immune cell infiltration in the cortico-meningeal compartment of MS patients, thus supporting the notion that localized inflammation within the meninges is implicated as a critical factor in the development of cortical demyelination in MS 170, 171. Further, these studies suggested that neuroinflammation was present in brain areas that were classified as normal-appearing white matter by conventional MRI 48. TSPO PET was capable of predicting later disease progression, pointing towards a potential prognostic role of tracers that allow early quantification of neuroinflammatory processes 172, 173. In addition to the potential prognostic role, TSPO PET has been employed to assess the efficacy of immunotherapy in MS. Indeed, TSPO PET corroborated the reduction in microglial activity in response to natalizumab treatment in the white matter of MS patients after 1 year of treatment 174. Similarly, the sphingosine l-phosphate receptor modulator, fingolimod, reduced microglial activation on TSPO PET in focal inflammatory MS lesions 175. However, it should be noted that a limited number of ten MS patients was included in each of the two studies, and thus these findings are to be treated with caution. Additional clinical trials have evaluated alternative TSPO-targeted PET ligands, including ^18^F-FEDAA1106 (NCT01031199), ^18^F-GEH120714 (NCT01738347), and ¹¹C-PK11195 (NCT02207075, NCT04239820), with a focus on safety, biodistribution, and signal quantification in both relapsing-remitting and progressive MS populations (**Table 1**). In addition, recent interventional trials have investigated the modulation of TSPO PET signals in response to anti-CD20 therapy (**Table 2**). For example, two studies are evaluating the effect of ocrelizumab on microglial activation using ^18^F-DPA-714 176 and ^11^C-PBR28 177, providing a framework to assess treatment-related changes in TSPO signal intensity and link them to structural and clinical outcomes (NCT03691077 and NCT04230174). In a recent consensus paper by the North American Imaging in Multiple Sclerosis (NAIMS) Cooperative, it was concluded that while MRI and TSPO-positive PET are emerging as potential biomarkers of chronic active lesions in MS, these biomarkers do not have equivalent sensitivity and specificity to histopathological findings. The latter was attributed to the lack of standardization in the use of currently available imaging measures for identification, quantification, and monitoring of these lesions 178. Nonetheless, preliminary studies have laid the foundation for standardized and adequately powered trials to come.

Despite the widespread use of TSPO PET, this class of probes is plagued by several shortcomings that are worthwhile mentioning. First, TSPO expression is not confined to microglia. Indeed, various studies have corroborated marked TSPO expression on perivascular, meningeal and choroid plexus macrophages and reactive astrocytes in the CNS 179-181. In a few instances, TSPO expression was reported on epithelial and endothelial cells, however, more studies are needed to corroborate the latter observation 181, 182. Another critical limitation of TSPO PET is that a number of clinically used probes are sensitive to a common polymorphism (rs6971) in the *TSPO* gene, imposing a need for genotyping and exclusion of the “low-affinity” variant prior to TSPO PET, which in itself may constitute a selection bias in clinical studies 163, 183, 184. It is currently debated whether the TSPO PET ligand, ^11^C-ER176 164, has the potential to allow for inclusion of all MS patients, independent of the *TSPO* gene variant. Another key limitation is the inability of TSPO-targeted probes to discriminate between pro- and anti-inflammatory microglia - information that can be crucial for instance to monitor response to anti-inflammatory therapy 185. Collectively, the limitations of TSPO PET have spurred the identification of novel biomarkers for the assessment neuroinflammation in humans.

In the past two decades, a number of promising targets for neuroinflammation imaging with PET have been identified, some of which are in exploratory preclinical stages and others that have been validated in humans 185. Among these targets, the cannabinoid receptor 2 (CB2), cyclooxygenase 1 and 2 (COX I and II), reactive oxygen species (ROS), colony stimulating factor 1 receptor (CSF1R) and purinergic receptors P2X7 and P2Y12, have been targeted with innovative PET probes that may be harnessed as MS-related biomarkers (**Figure 3**). Indeed, employing autoradiographic studies with brain sections of the EAE rat model and post-mortem human MS tissue, it was found that P2X7-targeted probes were associated with pro-inflammatory phenotype, while P2Y12 imaging was linked to an anti-inflammatory phenotype of innate immune cells in MS 186. A dual-tracer clinical study combining ^11^C-PK11195 with the P2X7-targeted ligand ^11^C-SMW139 (NCT04126772) is currently ongoing, aiming to assess microglial phenotypes and complement PET readouts with quantitative susceptibility mapping. Another approach leverages the visualizing ROS as a strategy for neuroinflammation imaging due to the enhanced oxidative stress in MS 187. ROS imaging with probes such as ^11^C-HM 188, ^11^C-DHQ1 189, ^18^F-ROStrace 190 or ^18^F-4FN 191 constitutes an intriguing concept to further develop our understanding of the role of ROS in the pathophysiology of MS. In particular, the ability to identify inflammatory foci was demonstrated for the PET reporter, ^18^F-4FN, which was employed to assess innate immunity activation in murine models of neuroinflammation 191. Along this line, CNS infiltration by myeloid cells was demonstrated on biopsy samples from patients with MS, which was corroborated by the ability of triggering receptor expressed on myeloid cells 1 (TREM1)-targeted PET with ^64^Cu-TREM1-mAb to monitor deleterious innate immune responses and disease progression in the EAE mouse model of MS 192. Another important target for neuroinflammation imaging is CB2, which is only marginally expressed in healthy brain tissues and was shown to be upregulated upon microglial activation in MS 193. Along this line, newly developed CB2 PET ligands including, but not limited to, thiazole-[194, 195], pyridine-[196-198], thiophen-[199] and oxoquinoline-[200-204]-based probes, could be of use in future studies assessing microglial activation in response to immunotherapy in MS. While the oxoquinoline derivative [^11^C]NE40 has been advanced to humans, clinical experience with [^11^C]NE40 confirmed brain penetration in humans, but did not detect the anticipated CB2R upregulation in Alzheimer's disease, highlighting challenges of CB2R-specificity and -selectivity in the CNS 205. More recent generations of pyridine-based CB2 tracers, such as [^18^F]RoSMA-18-d_6_, have demonstrated markedly enhanced affinity and selectivity in preclinical models and postmortem human ALS tissue, with efforts underway to advance them toward clinical evaluation 185. In parallel, the novel ligand [^11^C]MDTC has recently completed a first-in-human study, establishing safety, favorable dosimetry and test-retest reproducibility 206. CB2 PET ligands remain at an experimental stage, but next-generation candidates may ultimately enable sensitive *in vivo* assessment of microglial activation in MS patients. CB2 PET ligands are at an experimental stage and it remains to be seen whether these tools are of added value for diagnostic applications in animal models of MS and MS patients. While the COX I and COX II-targeted probes, ^11^C-PS13 207 and ^11^C-MC1 208, respectively, are currently tested in a phase II trial involving 16 MS patients (NCT05062083), the CSF1R-targeted PET with ^11^C-CPPC proved to be highly selective for microglial cells in rodents and non-human primates and may constitute another promising candidate to further refine neuroinflammation imaging in MS 209. Beyond the targets discussed above, additional clinical PET imaging studies are underway to explore alternative pathways in MS (**Table 1**). For instance, a clinical trial employing ^11^C-MRB (NCT03207464) is assessing norepinephrine transporter (NET) availability, which may reflect broader neuromodulatory disturbances 210. Immune cell tracking using ^89^Zr-oxine to label white blood cells (NCT03807973) represents an innovative strategy to capture systemic immune cell trafficking into the CNS 211, 212. Furthermore, a first-in-human study of ^11^C-BMS-986196 (NCT05064436) is exploring Bruton's tyrosine kinase (BTK) as a potential neuroinflammatory marker 213. Lastly, a multimodal PET trial employing ^18^F-florbetaben and ^18^F-DPA-714 (NCT05147532) seeks to combine myelin and TSPO imaging to assess lesion repair and immune activation longitudinally. These studies exemplify the expanding frontiers of translational molecular imaging in MS, paving the way for new mechanistic insights and imaging-enabled trial designs.

“The sphingosine-1-phosphate (S1P) signaling axis plays a central role in regulating immune cell trafficking, vascular integrity, and CNS homeostasis, and has emerged as a clinically validated therapeutic pathway in MS 214. Four oral disease-modifying therapies - fingolimod, siponimod, ozanimod, and ponesimod - are FDA-approved S1PR modulators that act by preventing lymphocyte egress from lymphoid tissues, thereby reducing CNS infiltration and neuroinflammation 120, 121. Given this therapeutic advent, there has been increasing interest in developing molecular imaging tools that can non-invasively quantify S1P receptor expression and drug-target engagement *in vivo*, thereby advancing translational research and informing clinical decision-making. Of note, S1P receptor subtype 1 (S1PR1) is widely expressed in lymphocytes, endothelial cells, oligodendrocytes, and neurons, rendering it particularly interesting for MS pathophysiology 215. Early PET tracers such as [^11^C]TZ3321 (also termed [^11^C]CS1P1) demonstrated selective binding to S1PR1 with high target-to-background contrast in preclinical models of vascular inflammation and EAE. Indeed, EAE rats showed increased uptake of [^11^C]TZ3321, which correlated with inflammatory lesions in the spinal cord, suggesting utility for monitoring neuroinflammation 216. Notably, first-in-human studies with [^11^C]CS1P1 have confirmed its safety, favorable dosimetry, as well as reproducible brain uptake in healthy volunteers. Further, PET studies have indicated the ability of [^11^C]CS1P1 to visualize S1PR1 expression in MS lesions, aligning with MRI-detected abnormalities, thereby highlighting the potential of [^11^C]CS1P1 PET to serve as a biomarker for disease progression 217-220. Building on these advances, a new generation of radiofluorinated S1PR1 tracers has been developed to overcome the logistical limitations of carbon-11. Radioligands such as [^18^F]TZ4877, [^18^F]FS1P1, and [^18^F]**6h** have demonstrated decent brain uptake and promising kinetics *in vivo* 221-224. Among these, [^18^F]TZ4877 showed high uptake in rat spinal cord lesions, while [^18^F]**6h** exhibited robust brain penetration in primates, positioning them as potential candidates for further development. Collectively, these tracers represent an important step toward enabling quantitative, target-specific imaging of S1PR1 expression in the CNS. Although less advanced, imaging efforts targeting other S1PR subtypes are underway. [^11^C]**5a**, a S1PR2-targeted probe, has shown uptake in pancreatic and immune tissues but limited brain penetration, prompting the development of various analogs with the aim to enhance brain uptake 225, 226. Novel radioligands for S1PR3 are at earlier stages of development, with preliminary fluorinated derivatives under evaluation. By contrast, S1PR5 is highly expressed on oligodendrocytes and natural killer cells, and has been implicated in promoting myelin preservation and neuroprotection 215. Given the approval of siponimod, which exhibits functional selectivity for S1PR5, developing PET tracers for this receptor has become particularly interesting - to assess remyelination dynamics and immune cell trafficking in MS 227. Taken together, S1PR-targeted molecular imaging represents a rapidly evolving field with high translational relevance for MS. PET radioligands for S1PR1 have progressed from preclinical validation to early human studies, while S1PR5-targeted imaging holds promise to monitor remyelination therapies. Looking ahead, integration of these approaches may not only refine patient stratification and therapeutic monitoring but also accelerate the development of next-generation S1P modulators with optimized CNS penetration and efficacy.”

## Targeting neurodegeneration and reactive astrogliosis

Molecular imaging with PET has been suggested to probe neuroaxonal degeneration in MS. Of note, a link between low ^18^F-fluorodexyglucose (^18^F-FDG) uptake in the grey matter and cognitive dysfunction in MS patients was established around the onset of the 21 century 228. The latter findings were later supported by a significant correlation of neuronal deterioration on magnetic resonance (MR) spectroscopy with attenuated ^18^F-FDG uptake in cortical and subcortical neural circuits that are relevant to cognitive function, including the hippocampus, cerebellum, caudate nucleus and prefrontal cortex 229. However, it should be pointed out that ^18^F-FDG uptake is not specific to neurons and can be affected by immune cells, with significant ramifications on the reliability of ^18^F-FDG PET for neurodegeneration imaging. Indeed, ^18^F-FDG uptake has been associated with neuroinflammation and it is well established that activated microglia significantly contribute to ^18^F-FDG brain uptake due to their enhanced demand for glucose 230. ^18^F-FDG PET in experimental autoimmune encephalomyelitis (EAE) - a common mouse model of MS - revealed that the ^18^F-FDG signal was associated with neuroinflammation. The authors observed that the ^18^F-FDG signal predicted the presence of neuroinflammatory lesions in the spinal cord and was sensitive to systemic immunosuppressive therapy 231. Collectively, these results suggested that ^18^F-FDG PET images can be significantly affected by changes in leukocyte metabolism, prompting calls for more selective probes to image neurodegeneration.

### Non-invasive assessment of neurodegeneration

As discussed above, attempts to visualize neurodegeneration involve probes that target SV2A. Indeed, given the ubiquitous expression of SV2A in presynaptic nerve terminals, these probes are capable of mapping synaptic density (**Figure 3**), providing valuable information on the progression of neurodegenerative disorders 139. While SV2A-selective PET ligands have been successfully translated to human studies and explored in neurodegenerative disorders, their application in MS is plagued by potential limitations. In particular, post-mortem investigations have yielded inconsistent findings regarding the relationship between synaptic density and MS pathology, raising concerns about the interpretability of SV2A PET signals in this context 137. Moreover, crude quantification of synaptic loss may lack specificity, as it does not discriminate between distinct synapse types that may be differentially affected in MS. To overcome these limitations, alternative molecular imaging strategies have been proposed to probe neurodegeneration with greater cellular or functional specificity. One such approach involves targeting gamma-aminobutyric acid (GABA) receptors 232. GABA is a naturally occurring neurotransmitter that is widely found in the CNS and plays a critical role in the pathophysiology of various neuropathologies, including MS, schizophrenia, epilepsy, anxiety and sleep disorder 233, 234. Notably, MR spectroscopy is not sensitive enough to distinguish between metabolic neurotransmitter concentrations and synaptic activity levels, whereas PET is more sensitive toward intra-synaptic changes, allowing for the quantification of GABA receptors across the entire brain 235. In particular, benzodiazepines have attracted much interest among the PET community, whereas the GABA_A_ receptor ligand flumazenil exhibited an outstanding target affinity and selectivity 236, 237. The latter has spurred research into radiolabeled flumazenil, ^11^C-flumazenil (^11^C-FMZ) and derivatives thereof as potential PET ligands for MS 232. Along this line, ^11^C-FMZ was evaluated in progressive and relapsing-remitting MS patients, displaying attenuated tracer uptake in both MS forms. Despite the small population size of 18 MS patients (9 progressive and 9 relapsing-remitting, these findings pointed toward a potential of ^11^C-FMZ to assess grey matter pathology in MS 238. In concert with these observations, a recently reported multi-ligand study found that binding of ^11^C-FMZ correlated with that of the TSPO radioligand, ^11^C-PK11195, suggesting that immune-mediated GABAergic alterations may contribute to the pathogenesis of MS 239. PET imaging was also used to show that neuronal damage is diffused throughout the cortical level in the grey matter of MS patients 238. By mapping neuronal damage in the grey matter of patients at different MS stages, the authors observed that ^11^C-FMZ binding was significantly decreased in the cortical grey matter. While these findings are considered promising, the use of GABA receptor-targeted PET in MS is its infancy and further studies in larger study populations are warranted to establish tangible clinical benefits that may arise from this approach.

Evidence to date points toward inflammation-linked generation of nitric oxide (NO) and reactive oxygen species (ROS) in MS, which contributes to mitochondrial dysfunction and attenuates local ATP production in demyelinated axons 240. Indeed, MS lesions are subject to a chronically reduced energy supply and enhanced energy demand by hyperexcitable demyelinated axons, prompting a state of energy deficiency and metabolic crisis 240-243. Since cells generate energy primarily through the mitochondria, mitochondrial proteins have emerged as appealing target to assess the energy balance in affected neurons and hence reveal early insights about detrimental molecular variables that are capable of predicting neurodegeneration. Among the five mitochondrial complexes, mitochondrial complex I (MC-I) exhibits the lowest activity and thus constitutes the rate-limiting factor in the regulation of oxidative phosphorylation 244. As such, MC-I targeted probes harbor potential to facilitate research into MS pathophysiology, potentially supporting the development of novel drug candidates that aim at restoring mitochondrial homeostasis in MS. To date, the most suitable MC-I PET ligand for brain imaging is ^18^F-BCPP-EF, which was first reported by Tsukada *et al* 244, 245. While MC-I PET has been employed in a series of neurological diseases 244, 246-250, this concept has yet to be explored in MS. Nonetheless, given the rapid progress in the field, which has yielded novel PET probes with excellent performance characteristics for MC-I imaging, it is anticipated that MC-I PET ligands may find near-term applications in MS research 245.

### Molecular imaging of reactive astrocytes

Astrocytes are involved at all stages of MS disease progression 251. Astrogliosis is a process in which astrocytes are activated and undergo a spectrum of molecular, cellular, and functional changes that occur in response to all forms and severities of CNS injury and disease 252. To date, astrogliosis constitutes an active ongoing field of research and molecular imaging of astrogliosis is at an exploratory stage. As such, applications of astrogliosis imaging in MS are limited. Of note, quantification of monoamine oxidase B (MAO-B), an enzyme located in the outer mitochondrial membrane, has been proposed as a potential target for visualizing astrogliosis 253. In post mortem brain specimens of eleven AD patients and five control subjects, the spatial distribution of catalytic sites for MAO-B was investigated by quantitative autoradiography, suggesting preferential expression in mitochondria of reactive astrocytes that were involved in neurodegenerative remodeling 253. Similarly, a recent first-in-human study with the highly selective MAO-B PET ligand [^18^F]SMBT-1 suggested that the tracer can potentially be used as a surrogate marker for astrogliosis 254. Other MAO-B targeted PET ligands such as [^18^F]THK5351 and [^11^C]deuterium-L-deprenyl have been tested in MS patients and were found to add incremental value by identifying reactive astrocytes in a subset of inactive MS lesions 255, 256. Beyond these ligands, early human studies with [^11^C]L-deprenyl and its structural analog [^11^C]DED confirmed retention in MAO-B-rich brain regions and increased uptake in early AD and MCI 257-259. More recently, the reversible inhibitor [^11^C]SL25.1188 has been validated in first-in-human studies, offering improved quantification, favorable kinetics, and reduced susceptibility to confounding radiometabolites 260. Collectively, these developments underscore the translational potential of MAO-B-targeted imaging biomarker of astrocyte reactivity in MS and related neurodegenerative conditions. Another strategy to image astrocytes involves imaging of metabolic or exocytotic mechanisms in reactive astrocytes. For instance, metabolic activity can be estimated with carbon-11 labeled acetate (^11^C-acetate). It is currently believed that ^11^C-acetate uptake in the brain can mainly be attributed to metabolism in astrocytes 261. Along this line, a study found that ^11^C-acetate uptake was higher in the brains of MS patients, as compared to healthy controls, indicating an enhanced activation of astrocytes in the patient cohort 262, 263. Another more exploratory concept toward imaging astrocytes includes targeting the adenosine A2A purinergic receptor (A2AR) with ^11^C-TMSX 264. Indeed, ^11^C-TMSX uptake was increased in the normal-appearing white matter in secondary progressive MS patients and was correlated with neuroaxonal damage 265. Further studies are warranted to assess the validity of A2AR as a target for astrogliosis imaging in MS. Overall, astrocyte PET is still in its infancy, however, the fundamental role of astrocytes in the pathophysiology of MS has been established from preclinical and post-mortem studies and the availability of suitable clinical PET ligands has potential to facilitate contemporary research and clinical care for MS patients. In particular, the concept of imaging astrogliosis via MAO-B seems promising based on the currently available body of literature.

## Advanced magnetic resonance imaging techniques

Although PET imaging provides a decent combination of molecular specificity and quantitative accuracy, its broader clinical deployment remains limited by radiation burden to the patients and access to radiopharmaceutical skills and infrastructure. In contrast, advanced MRI techniques have emerged as non-radioactive alternatives in order to address the challenge of interrogating key molecular and microstructural changes in MS. One example is MTI 47, 48, which measures the exchange of magnetization between protons in free water and protons bound to macromolecules such as myelin. Another is DTI 49-51, which provides information on tissue microstructure by quantifying the local diffusion tensor of water. Diffusion MRI methods are increasingly being evaluated for clinical application in MS, particularly when combined with biophysical models such as neurite orientation dispersion and density imaging (NODDI), where the total diffusion signal is modeled as a sum of intra-neurite and free water compartments 266. These approaches have not only focused on white matter but have also been extended to study cortical gray matter. Recently, a novel diffusion MRI model, soma and neurite density imaging (SANDI), has been applied to clinical data to investigate inflammation and degeneration in the cortical tissue 267. Recent developments also include the introduction of novel hardware for ultra-high gradient strength diffusion MRI, such as the Connectome 2.0 scanner with gradients up to 500 mT/m 268, and the application of machine learning approaches to improve diffusion data fitting 269. Quantitative T1 (qT1) mapping has also been explored as a sensitive measure of tissue microstructure, particularly in detecting cortical demyelination and remyelination. Recent postmortem studies demonstrated that qT1 correlates with histopathological markers of myelin and can differentiate between demyelinated, remyelinated, and normal-appearing cortical areas, underscoring its potential as a biomarker of tissue repair in MS 270. Importantly, multimodal strategies that combine different qMRI techniques, for example, MTI with diffusion-based measures or MTI with quantitative susceptibility mapping (QSM), have shown promise in enhancing biological specificity, improving lesion characterization, and enabling a more comprehensive assessment of demyelination and repair dynamics 271. It should be noted, however, that the reproducibility of some of these approaches remains to be validated, as studies are often conducted at different field strengths, with scanners from various vendors, as well as the use of non-standardized acquisition parameters. Another promising set of approaches are based on QSM, which measures local magnetic susceptibility to assess the spatial distribution of both paramagnetic sources (e.g., iron-containing compounds such as ferritin and hemoglobin) and diamagnetic sources (e.g., myelin and calcium) 272. QSM has been particularly successful in identifying paramagnetic rim lesions (PRLs), a subset of chronic active lesions characterized by iron deposition in activated microglia/macrophages 273. More recently, a technique known as χ-separation has been developed, enabling independent mapping of diamagnetic (myelin) and paramagnetic (iron) contributions to tissue susceptibility 274. This method has been validated in relapsing-remitting MS patients, where concurrent monitoring of both demyelination and inflammation in the same voxel was demonstrated 275. Building on this, it is now possible to acquire combined QSM and MTI data in a single MRI acquisition 276, further illustrating the versatility of these techniques. Nonetheless, it is important to note that most χ-separation studies to date have been performed on high-field research scanners, which are not widely available in routine clinical practice. In addition to these tools, sodium-23 MRI (^23^Na MRI) has also been explored in MS. The underlying principle is that sodium channels become abundantly available in demyelinated axons, leading to an abnormal accumulation of sodium within lesions. Indeed, increased total sodium concentration and intracellular sodium volume fraction have been shown to correlate with lesion size as well as with clinical measures of disability 277. Moreover, ^23^Na MRI parameters have been reported to correlate with NODDI-derived diffusion metrics in the same patients 278. Despite these advances, several challenges remain. The degree of histopathological specificity is still limited, complicating the quantitative analysis of demyelination with MRI 31, 44. Reproducibility and widespread clinical availability also remain concerns, since many of these approaches rely on sophisticated biophysical modeling and state-of-the-art MRI equipment 51. For ^23^Na MRI, additional hurdles include low signal-to-noise ratio due to the much lower gyromagnetic ratio of sodium compared to protons (3.8-fold lower), which leads to longer acquisition times and reduced spatial resolution. Another emerging concept relies of ^19^F-based MRI detection. Indeed, applications of ^19^F-MRI have demonstrated the ability to directly visualize and quantify fluorinated small molecules, including the FDA-approved S1PR modulator siponimod. In a proof-of-concept study, it was demonstrated that fluorine MRI is capable of mapping the biodistribution of siponimod in mice, revealing quantitative readout across the stomach, liver, kidney, thymus and the brain 279. Using optimized ultrashort echo time sequences, siponimod was found to preferentially distribute to the cerebrum, with heterogeneous regional distribution patterns highlighting that ^19^F-MRI can capture not only CNS penetration but also spatial distribution patterns in drug accumulation. Importantly, sensitivity modeling indicated that acquisitions at standard 3.0 T clinical MRI systems could achieve ~2.75-fold higher relative sensitivity compared to preclinical 9.4 T setups. The study also emphasized that fluorine MRI offers the unique advantage of imaging unmodified drugs at clinically relevant doses without ionizing radiation, thereby distinguishing it from PET approaches that require radiolabeling. Collectively, these findings highlight a promising strategy for monitoring CNS biodistribution, pharmacokinetics, and open the avenues for theranostic applications of fluorinated MS therapies. While still at early stages, fluorine MRI represents an important addition to the molecular imaging arsenal, particularly for longitudinal safety studies and drug development pipelines where accessibility and non-radioactive biomarker options are critical. Taken together, these advanced MRI methods provide important insights into MS pathology. Similar to PET, MRI-based applications can interrogate physiological and pathophysiological processes, enabling quantitative assessment of molecular events. Given the diversity of applications, PET and MRI should be considered complementary modalities, with hybrid PET-MRI representing a particularly intriguing avenue for paired applications in clinical applications.

## Concluding remarks

Notwithstanding the significant advances in the diagnosis and management of MS over the past two decades, limitations of contemporary clinical routine include a complex diagnostic workup that often requires sophisticated differential diagnoses, as well as the lack of effective tools to predict the course of disease and select the most effective treatment plan. Molecular imaging with PET is increasingly used in preclinical and clinical research - with the aim to overcome these limitations, however, these molecular imaging tools have not yet been implemented in clinical MS guidelines to date. While the potential of myelin-directed imaging probes for monitoring disease progression and response to therapy has been demonstrated, TSPO PET has shown initial encouraging results as a prognostic marker, which holds promise to improve clinical decision-making by allowing early clarification of the optimal therapeutic strategy, particularly for fast-progressing forms of MS. Other high-potential applications include the assessment of synaptic density and astrocyte reactivity in MS, however, more clinical data is required to corroborate the utility of these exploratory tools. Similarly, the non-invasive assessment of molecular alterations at the BBB holds potential to serve as an early marker in MS. These alterations may involve P-glycoprotein (P-gp) and multidrug resistance-associated proteins (MRPs), which are highly abundant at the BBB. Alterations in the composition of the BBB by such efflux proteins are typically reflected as shifts in the brain uptake kinetics of probes that are routinely used in the clinic 280. The BBB integrity can further be assessed through advanced cerebral perfusion imaging techniques that utilize oxygen-15-labeled water, as well as a range of ionized PET and SPECT ligands. These radiolabeled ligands demonstrate a markedly increased uptake into brain tissues in response to compromised BBB permeability 281.

While structural and anatomical MRI remain firmly established as routine tools in clinical diagnosis and management of MS, thereby providing high-resolution images of CNS lesions, more advanced MRI techniques have been suggested to probe molecular and microstructural processes such as myelin integrity and iron deposition. These approaches are increasingly applied in research settings and may gradually transition into clinical use as protocols are standardized and validated. While inferior to MRI in terms of spatial resolution, PET offers high sensitivity and allows the use of tracer doses that are typically below a pharmacological threshold 30. This makes PET particularly suitable for quantitative studies of drug biodistribution, receptor occupancy, and pharmacodynamic effects, which can directly inform dose selection in clinical development. Moreover, established modeling frameworks allow for absolute quantification when a metabolite-corrected input function or validated reference region is available. At the same time, it is important to note that PET comes with practical and regulatory challenges, including the need for on-site or nearby radiochemistry infrastructure, the limited half-lives of commonly used isotopes, moderate spatial resolution, and a radiation burden that may constrain repeated applications (**Table 3**). MRI does not share these limitations, which contributes to its wide availability and suitability for repeated longitudinal studies. Nonetheless, MRI often requires higher concentrations of contrast agents, and establishing modeling workflows for quantitative molecular imaging with MRI remains challenging. Thus, while MRI is expected to remain the clinical mainstay for structural and functional assessments, PET is likely to gain importance where highly sensitive and quantitative molecular readouts are warranted. Looking ahead, the convergence of PET with advanced MRI techniques, along with integrative analytic platforms, is poised to transform the diagnostic and therapeutic landscape of MS. The incorporation of multi-omics datasets (genomic, transcriptomic, and proteomic) with imaging-derived biomarkers, supported by artificial intelligence-driven analytical platforms, will enable more precise patient stratification, dynamic monitoring of disease progression, and prediction of therapeutic response. Such convergence is envisioned to support patient stratification, therapeutic monitoring, and biomarker-driven trial design, while also contributing to the future use of molecular imaging tools in the routine clinical management of MS.

## Figures and Tables

**Table 1:** ** Selected diagnostic clinical trials with radioligands used in multiple sclerosis.** Molecular targets, radioligands used, primary and secondary endpoints - sorted by date in decreasing order. *Abbreviations: TEAE: Treatment-emergent adverse event;. QSM: Quantitative susceptibility mapping; SUV: Standardized uptake values; EDSS: Expanded disability status scale; SPMS: Secondary progressive multiple sclerosis; rrMS: Relapsing and remitting MS; VT: Tissue volume of distribution; NfL: Neurofilament Light chain; TSPO: Translocator protein; NET: Norepinephrine transporter; GABAA: Gamma-aminobutyric acid receptor A; SV2A: Synaptic vesicle glycoprotein 2A.*

Clinical trial	Target	Radioligand	Primary end points	Secondary end points	Study dates
NCT01031199 (Phase I)	TSPO	^18^F-FEDAA1106 (BAY85-8101)	Standard quantification variables from 3D PET and brain modeling.	Evaluated safety parameters, including TEAEs, electrocardiograms and vital signs	2009 - 2009
NCT01738347 (Phase I)	TSPO	^18^F-GEH120714	Evaluated safety of ^18^F-GEH120714 Injection in healthy subjects and subjects with rrMS	Determined biodistribution, internal radiation dosimetry and effective dose in healthy volunteers.	2013 - 2016
NCT01651520 (Early Phase)	GABA_A_	^11^C-Flumazenil	Changes in neuronal atrophy and EDSS	n/a	2013 - 2019
NCT02207075 (Early Phase)	TSPO	^11^C-PK-11195	Baseline and changes of whole brain uptake of ^11^C-PK-11195 in SPMS subjects.	Relationship between T2-hyperintensities, conventional MRI measures and whole brain ^11^C-PK-11195 PET	2014 - 2020
NCT02649985 (Phase I & II)	TSPO	^18^F-PBR06 and ^11^C-PBR28	Measured VT levels	Measured SUVR values	2016 - 2021
NCT03207464 (Phase I & II)	NET	^11^C-MRB	Measured VT levels	n/a	2017 - 2021
NCT04126772 (Early Phase)	TSPO and P2X7	^11^C-PK11195 ^11^C-SMW139	^11^C-PK11195 binding, ^11^C-SMW139 binding and QSM signal in MS patient brains	Binding and QSM signal in brains of healthy controls, as well changes in clinical MS metrics	2019 - 2025
NCT04239820(Early Phase)	TSPO	^11^C-PK11195	Changes in microglia-activity in MS patients measured by ^11^C-PK11195	MRI metrics, EDSS, MS composite score, changes in serum neurofilament light (NfL) and glial fibrillary acid protein (GFAP), QSM signal in MS patient brain.	2020 - 2024
NCT04634994 (Early Phase)	SV2A	^18^F-SDM8 (^18^F-SynvesT-1)	Calculated VT over various brain regions	Calculated SUV over various brain regions	2021 - 2022
NCT04699747 (Phase I)	Potassium channels	^18^F-3F4AP	Comparison of binding for ^18^F 3F4AP in the brain of healthy volunteers and MS subjects, as well as measure TEAEs.	Comparison of binding for ^18^F 3F4AP in brain lesions of multiple sclerosis subjects and determine variability in healthy controls and multiple sclerosis subjects	2021 - 2024
NCT04710550 (Phase I)	Potassium channels	^18^F-3F4AP	Number of subjects with TEAEs	n/a	2021 - 2025
NCT03807973 (Phase I)	White blood cells	^89^Zr-oxine	SUVs for the patients and healthy controls in various brain regions.	n/a	2021 - 2025
NCT05064436 (Phase I)	Bruton's tyrosine kinase	^11^C-BMS-986196	Incidence of TEAEs	Calculated SUV and VT in the brain	2021- 2023
NCT05147532 (Early Phase)	Myelin, TSPO	^18^F-Florbetaben ^18^F-DPA-714	Proportion of lesional demyelinated voxels at baseline that undergo remyelination during the relapsing and the progressive phases of MS	Percentage of voxels classified as significantly activated compared to control white matter, and the number/proportion of activated MS lesions measured via ^18^F-DPA-714 PET	2022 - 2024

**Table 2:** ** Selected target occupancy and therapy monitoring trials with probes used in multiple sclerosis.** Molecular targets, radioligands used, primary and secondary endpoints - sorted by date in decreasing order. *Abbreviations: TEAE: Treatment-emergent adverse events; MTR: Magnetization transfer ratio; QSM: Quantitative susceptibility mapping; SUV: Standardized uptake values; EDSS: Expanded disability status scale; VT: Tissue volume of distribution; WM: White matter; NfL: Neurofilament light chain; TSPO: Translocator protein; COX: Cyclooxygenase.*

Clinical trial	Target	Ligands	Primary end points	Secondary end points	Study dates
NCT03691077 (Phase III)	TSPO	Ocrelizumab, 18F-DPA714 and MRI	Determined if ocrelizumab treatment is associated with a decrease in the extent of microglial activation in WM	Decreased of microglial activation as measured by 18F-DPA-714 PET in various brain regions	2018 - 2024
NCT04230174 (Phase IV)	TSPO	11C-PBR28, Ocrelizumab	Measured changes in 11C-PBR28 uptake in MS patient brains under Ocrelizumab therapy, incl. WM lesion load, cortical atrophy and demyelination in the cortex and in the NAWM as measured by MTR	Whether changes in 11C-PBR28 uptake or in structural imaging metrics correlate with measures of neurological disability and cognition	2020 - 2022
NCT04941781 (Phase I)	Muscarinic acetylcholine receptor M1	11C-PIPE-307	M1AChR receptor occupancy determined from VT of 11C-PIPE-307	n/a.	2021 - 2021
NCT05062083 (Phase II)	COX-1/ COX-2	11C-PS13 and 11C-MC1	SUVR of Lesions with Injection of 11C-PS13 or 11C-MC1, before and after blockade with Ketoprofen or Celecoxib	SUVR of Chronic Active Lesions with Injection of 11C-PS13 and 11C-MC1	2022 - 2024
NCT06292923 (Phase II)	CD3, TSPO	Foralumab and 18F-PBR06	(i) Incidence of TEAEs in patients (ii) Changes in the Total Nasal Symptom Score (TNSS). Changes in microglial activation baseline measured via 18F-PBR06 PET.	(i) Changes in EDSS and other clinical scores. (ii) Changes in the mean number of gadolinium-enhancing lesions per T1-weighted MRI scan, and paramagnetic rim lesions via QSM.	2023 - 2024
NCT06083753 (Phase II)	Muscarinic acetylcholine receptor M1	PIPE-307 MTR imaging	Number of patients with TEAE as well as Change in binocular 2.5% low contrast letter acuity (LCLA)	(i) Therapeutic efficiency via various tests of visual and physical impairment in MS patients. (ii) change in MRI measures of myelination and MS disease activity, NfL and blood concentration levels of PIPE-307	2023 - 2025
NCT05849467 (Phase I)	CD8+ T cell	89Zr-Df-crefmirlimab	Detected and localized infiltration of CD8+ T cells in the CNS of adults with MS and PML.	Assess safety of 89Zr-Dfcrefmirlimab in participants with CNS disease, and compare SUV before and after immune reconstitution, either spontaneous or facilitated	2023 - 2025
NCT05849467 (Phase I)	CD8+ T cell	89Zr-Df-crefmirlimab	Detected and localized infiltration of CD8+ T cells in the CNS of adults with MS and PML via PET-CT scans	Assess safety of 89Zr-Dfcrefmirlimab, and compare SUV before and after immune reconstitution, either spontaneous or facilitated	2023 - 2025
NCT06330077 (Phase II)	myelin	Ifenprodil and 18F-florbetaben	Efficacy of ifenprodil on neural remyelination	Effect of ifenprodil treatment on remyelination levels and axonal damage in the visual pathway assessed by the amplitude of P100 and the blood concentration of NfL	2024 - 2027

**Table 3:** ** Comparison of positron emission tomography (PET) and magnetic resonance imaging (MRI) with a specific focus on multiple sclerosis (MS).** While each modality has distinct advantages and limitations. Hybrid PET/MRI applications hold potential to enhance trial design and therapy monitoring in MS.

	Positron Emission Tomography	Magnetic Resonance Imaging
Sensitivity & resolution	Offers high sensitivity - typically enables tracer use at microdosing, which minimizes pharmacological interference, limited resolution compared to MRI.	Lower molecular sensitivity but widely used in MS for detecting lesions. Advanced protocols are being explored to probe inflammatory or myelin dynamics, high spatial resolution.
Quantification of molecular processes	Supported by established kinetic modeling frameworks that allow absolute quantification when appropriate input functions are available, directly applicable to MS drug development pipelines.	Quantitative MRI methods (e.g. myelin water imaging, quantitative susceptibility mapping) are increasingly applied in MS research to assess myelin integrity, iron deposition, and biochemical changes, though standardization remains a challenge.
Drug development utility	Particularly valuable in MS for visualizing myelin dynamics and neuroinflammatory targets (e.g. TSPO, P2X7, MAO-B), and for informing dose selection by quantifying CNS penetration and target occupancy of novel drug candidates.	Central to MS clinical trials as the established tool for lesion load, brain atrophy, and treatment monitoring. Advanced MRI applications (e.g. magnetization transfer, diffusion-based metrics) are being evaluated as novel biomarkers in MS.
Infrastructure & logistics	Requires radiochemistry facilities and radiotracer availability; the short half-life of commonly used isotopes limits broad accessibility. Radiation exposure may constrain repeated use, especially in younger MS populations.	No radiation burden and no need for tracer production, which has enabled MRI to become the routine imaging tool in MS. Longitudinal imaging of lesion evolution and cortical anatomy is readily feasible.
Clinical readiness	Molecular PET applications in MS remain mainly research-focused, though myelin-targeted tracers and receptor targeted ligands have entered clinical arena for MS-related applications. For instance, TSPO PET is widely used in MS research to study microglial activation.	Conventional MRI is standard of care for MS diagnosis and monitoring. Molecular MRI applications are largely investigational but may gradually transition into clinical use as validation increases.
Complementarity	Target-specific molecular readouts can complement MRI-derived lesion and atrophy measures.	Provides high-resolution anatomical, structural, and functional information that contextualizes PET signals and adds information on lesion burden and progression.
Integration	Hybrid PET/MRI allows simultaneous assessment of MS pathology at molecular, structural, and functional levels, exemplifying how multimodal imaging may support precision monitoring and biomarker-driven trial design.	
